# Stretchable, Multiplexed, and Bimodal Sensing Electronic Armor for Colonoscopic Continuum Robot Enhanced by Triboelectric Artificial Synapse

**DOI:** 10.1002/adma.202502203

**Published:** 2025-06-05

**Authors:** Yuyang Sun, Tao Chen, Dongsheng Li, Hanyang Li, Tianci Ji, Fengxia Wang, Lining Sun, Chengkuo Lee, Huicong Liu

**Affiliations:** ^1^ School of Mechanical and Electrical Engineering Jiangsu Key Laboratory of Embodied Intelligence Robot Technology Soochow University Suzhou 215006 China; ^2^ School of Future Science and Engineering Soochow University Suzhou 215200 China; ^3^ Department of Electrical & Computer Engineering National University of Singapore Singapore 117576 Singapore

**Keywords:** colonoscopic continuum robot, full‐coverage, multiplex, strain sensing, stretchable electronic armor, tactile sensing, triboelectric encoding

## Abstract

Colonoscopic continuum robots often lack sensing capabilities, risking tissue damage. An ideal robot electronic skin should offer full‐body coverage, multiplexing, stretchability, and multifunctionality, but integration is challenging due to the robot's elongated structure. This work presents a stretchable electronic armor (E‐armor) with a 3D crosslinked structure that enables 300 mm full coverage while accomplishing multiplexed simultaneous tactile and strain sensing through bioinspired artificial synapse mechanisms. The E‐armor integrates 48 tactile sensing points through bilayer co‐electrode strategy, reducing wiring while combining triboelectric encoding intelligence with innovative stretchable triboelectric interlinked films (TIFs) to form a triboelectric artificial synapse that generates digitally encoded signal pairs upon contact. A convolutional neural network and long short‐term memory network (CNN‐LSTM) deep learning framework achieve 99.31% accuracy in identifying multi‐point tactile signals. A sodium alginate/polyacrylamide/sodium chloride (SA/PAM/NaCl) conductive hydrogel serves as a strain sensing element, providing excellent stretchability and biocompatibility, and allowing precise inference of bending angles at 12 strain sensing edges. A compliance control strategy coordinates tactile and strain signals to autonomously adjust continuum robot postures while ensuring smooth operation. The digital twin‐based 3D visualization interface enhances human–robot interaction by digitally reconstructing both tactile and strain feedback, enabling real‐time visualization of the continuum robot's intracolonic posture.

## Graphical abstract

Colonoscopic continuum robots lack sensing, endangering intestinal tissue. An ideal sensing array is hard to integrate. This work introduces a 3D crosslinked stretchable E‐armor with full‐coverage and multiplexing. It has bimodal sensing, forms a triboelectric synapse, uses CNN‐LSTM, and has a hydrogel and innovative triboelectric materials. A control strategy and digital twin interface enhance safety and interaction in colonoscopy.

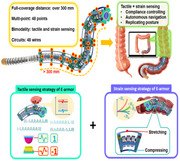

## 1 Introduction

Colorectal cancer (CRC) is the third leading cause of cancer‐related deaths worldwide, with over 1.85 million new cases diagnosed annually.^[^ ^1^ ^]^ Early diagnosis and treatment of CRC through routine colonoscopy are critical for improving patient survival rates.^[^ ^2^ ^]^ During the colonoscopic examination, operative safety typically depends on the surgeon's tactile experience and visual feedback from the endoscopic camera, which may increase the risk of complications such as peritonitis and intestinal bleeding.^[^ ^3^ ^]^ Robot‐assisted colonoscopy offers significant advancements in autonomous posture adjustment and operational precision, potentially enhancing both the accuracy and safety of these procedures.^[^ ^4^, ^5^, ^6^, ^7^, ^8^, ^9^ ^]^ Inspired by mollusks and flexible organisms, such as snakes,^[^ ^10^, ^11^, ^12^ ^]^ elephant trunks,^[^ ^13^, ^14^, ^15^ ^]^ and octopus arms,^[^ ^16^, ^17^, ^18^, ^19^ ^]^ continuum robots have attracted increasing attention^[^ ^20^, ^21^, ^22^, ^23^ ^]^ and are particularly suited for navigating the complex, confined environment of the human colon. The internal structure of the intestine is convoluted, comprising narrow lumen, angulated segments, and numerous folds; hence, colonoscopic continuum robots must ensure gentle contact with the intestinal wall and continuously adjust their posture to prevent frictional damage, bleeding, or perforation.^[^ ^3^ ^]^ As such, the development of tactile and strain sensing capabilities, along with real‐time feedback mechanisms, is essential for ensuring safe and effective robot‐assisted colonoscopy.

To perceive the strain of continuum robots, various sensors have been developed based on principles, such as the Fiber Bragg Grating (FBG),^[^ ^24^ ^]^ electromagnetism,^[^ ^25^, ^26^ ^]^ Hall effect,^[^ ^27^ ^]^ and stretchable optical waveguides.^[^ ^28^ ^]^ However, these technologies face several limitations, including high costs and limited bending angles in FBG sensors, susceptibility to magnetic interference in electromagnetic and Hall effect sensors, and complex integration requirements of optical waveguide sensors, rendering them unsuitable for colonoscopic continuum robots.^[^ ^29^ ^]^ Drawing inspiration from the sensory functions of animal skin, studies have developed flexible and stretchable electronic skins (E‐skins) embedded with artificial synapses and showing considerable promise in various applications, including wearable electronics,^[^ ^30^, ^31^, ^32^, ^33^, ^34^, ^35^ ^]^ medical health monitoring,^[^ ^36^, ^37^, ^38^, ^39^ ^]^ human–machine interfaces,^[^ ^38^, ^40^, ^41^, ^42^, ^43^, ^44^, ^45^ ^]^ and intelligent robotics.^[^ ^46^, ^47^, ^48^, ^49^ ^]^ Given the multi‐degree of freedom (multi‐DOF) flexible structure of continuum robots and the need for large‐area full‐coverage across all joints, the stretchability and stability of the sensing elements materials used in E‐skins are key research priorities. Flexible and stretchable conductive materials have gained prominence in recent years. In addition to traditional carbon‐based materials,^[^ ^50^, ^51^, ^52^ ^]^ liquid metals such as eutectic gallium–indium and gallium–indium–tin alloys have gained attention in E‐skin research owing to their fluidity and high conductivity. These liquid metals can be injected into elastomer microchannels or used in 3D printing to fabricate flexible strain sensors.^[^ ^53^, ^54^, ^55^, ^56^ ^]^ However, liquid metals pose a risk of leakage, which is a critical limitation for biomedical applications as they do not meet the non‐toxic requirements necessary for intestinal procedures. In contrast, ionic conductive hydrogels, recently developed through material innovations, offer a compelling alternative. These hydrogels are soft, highly stretchable, and inherently biocompatible with programmable mechanical adaptability. Importantly, they can form stable, adhesive interfaces with elastomers without leakage, effectively overcoming the stability challenges faced by conventional materials.^[^ ^57^, ^58^, ^59^ ^]^ Hence, they are highly promising candidates for use in wearable electronics and medical robotics, particularly in applications where safety and environmental resilience are essential. Stretchable adhesive hydrogel‐based strain sensors can significantly enhance the perceptive capabilities of continuum robots, enabling closed‐loop control and real‐time motion monitoring.^[^ ^60^ ^]^

In recent years, there has been a growing interest in developing tactile perception in continuum robots.^[^ ^61^ ^]^ For example, a dual‐mode intelligent skin has been designed to respond to both tactile and non‐contact stimuli, facilitating complex motion controls and distance sensing.^[^ ^62^ ^]^ Ring‐shaped sensors have also demonstrated effective collision‐detection capabilities during operation.^[^ ^63^, ^64^ ^]^ In addition, a stretchable pressure sensor inspired by Shar–Pei dog skin has been developed that employs reduced graphene oxide electrodes with a biomimetic structure to improve the collision detection ability in narrow spaces.^[^ ^65^ ^]^ However, most of these sensors cover relatively small sensing areas and incorporate a limited number of sensing points. Moreover, achieving full‐coverage with multimodal sensing arrays can result in excessively high circuit density, posing significant integration challenges within the constrained spaces of continuum robots.^[^ ^66^, ^67^ ^]^ Tactile sensing arrays based on single‐electrode triboelectric nanogenerators (TENGs) offer a promising solution. These sensors are self‐powered, highly flexible, and structurally adaptable, rendering them particularly well‐suited for continuum robots. Importantly, their self‐powered nature eliminates the risks of tissue damage from electrical leakage during contact with intestinal tissue, a critical consideration for safe biomedical applications. The inherent flexibility and stretchability of TENG‐based sensors ensure reliable integration into multi‐DOF continuum robots, maintaining tactile functionality even under substantial deformation. Further, customizable shared electrode configurations reduce wiring complexity in sensor arrays, enabling deployment within the narrow, confined spaces typical of continuum robots. These combined advantages, including safety, mechanical compatibility, and scalable integration, render TENG‐based sensors particularly well‐suited for achieving full‐body tactile perception in minimally invasive surgical robots. However, shared electrode designs face notable limitations. Specifically, they struggle to accurately localize contact positions,^[^ ^68^, ^69^, ^70^ ^]^ particularly when multiple contact points occur simultaneously. In addition, many single‐electrode TENG sensing arrays use a shared substrate, which can result in charge drift and signal crosstalk between sensing units, ultimately compromising sensing accuracy and reliability.

**Figure 1:**
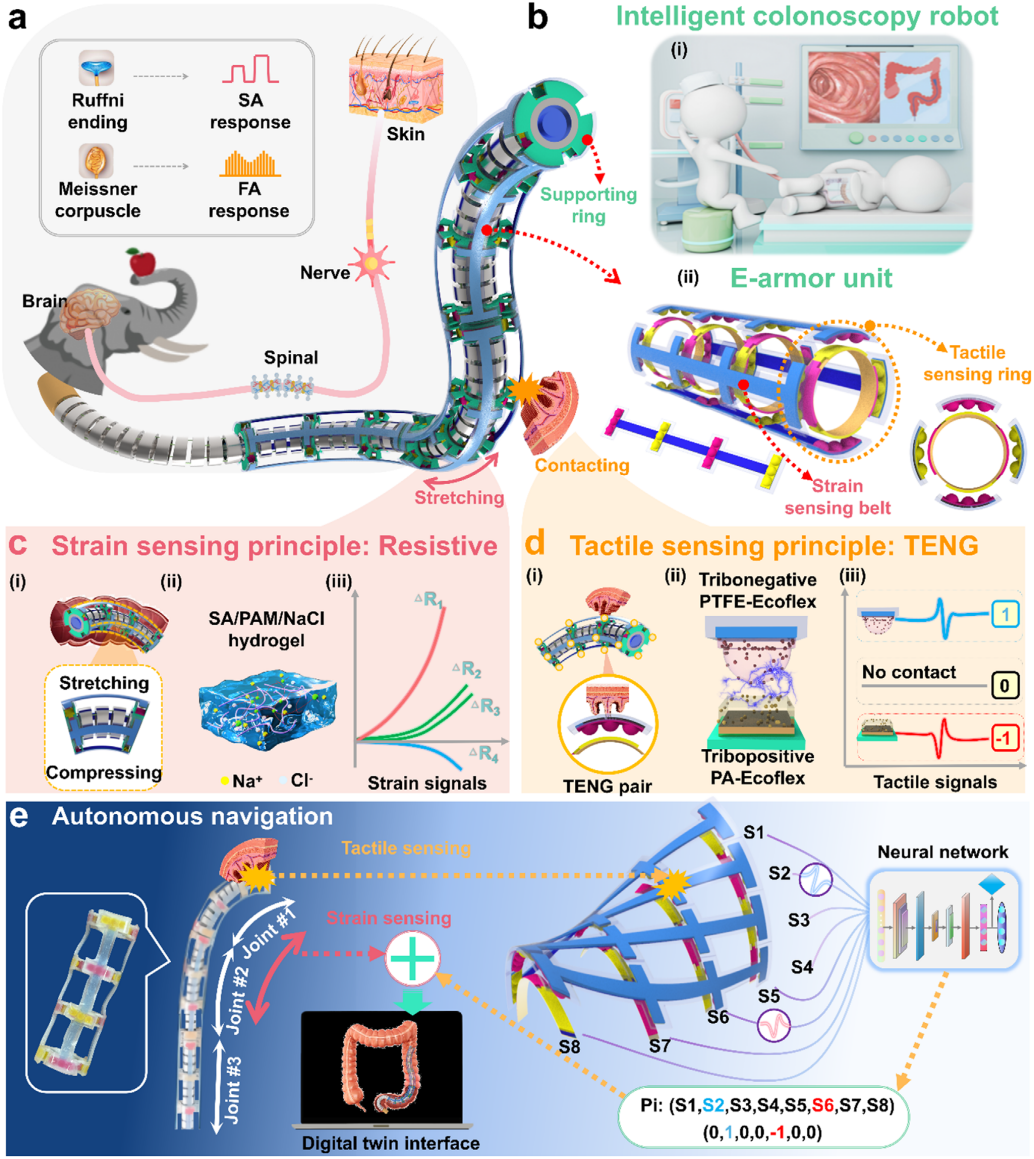
Proposed E‐armor based on a bimodal and triboelectric encoding strategy for colonoscopy examinations. a) Schematic illustration of multiple sensory receptors in animal skin for tactile perception. b‐i) Concept of an intelligent colonoscopic continuum robot. b‐ii) Schematic of the E‐armor unit structure. c) Strain sensing strategy of the E‐armor. c‐i) Schematic of the strain sensing net. c‐ii) Structure of the SA/PAM/NaCl hydrogel. c‐iii) Strain signal output and posture perception strategy of the continuum robot. d) Tactile sensing strategy of the E‐armor. d‐i) Schematic of the tactile sensing net. d‐ii) TENG effect between PTFE‐Ecoflex and PA‐Ecoflex. d‐iii) Tactile signal output and digital encoding strategy of the E‐armor. e) Autonomous navigation of the continuum robot with integrated E‐armor, incorporating bimodal information closed‐loop feedback.

To address these challenges, bioinspired approaches offer valuable design cues. For example, the structure and sensory capabilities of an elephant's trunk, where the animal's somatosensory network delivers precise sensory information through receptors located in the trunk's skin, provide a compelling model for developing efficient tactile systems. Some of these receptors, such as slow‐adapting (SA) Ruffini endings and fast‐adapting (FA) Meissner corpuscles receptors, can detect stretching and vibrations of the skin.^[^ ^71^, ^72^ ^]^ Sensory signals from these receptors are transmitted via nerves to the brain, where they are interpreted to perceive information, such as limb bending and touch intensity (**Figure** **1**a). In a similar manner, a colonoscopic continuum robot with multi‐DOF structure could also be made capable of surface tactile perception and posture bending detection when operating inside the human intestine. These sensing abilities will enable the robot to detect contact with intestinal tissues during medical procedures and adjust its posture in real time, thereby ensuring safe and effective examinations. This work introduces a compact, bimodal, and stretchable electronic armor (E‐armor) that integrates both tactile and strain sensing functions within the confined space of continuum robots, enabling advanced perception and control in minimally invasive surgical applications. Through a 3D cross‐linked sensing net architecture requiring only 48 wires, it achieves a full‐coverage sensing over a length exceeding 300 mm, incorporating 48 tactile sensing points and 12 strain sensing edges. This design addresses the critical challenge of structural integration in elongated robotic systems. All materials used in the E‐armor are biocompatible, meeting the non‐toxicity requirements essential for safe operation within the intestinal environment. Each sensing unit incorporates an ionic conductive hydrogel that functions both as a strain‐sensing element and a flexible electrode. Based on the resistive sensing principle, the hydrogel generates continuous and smooth strain signals that mimic the stimulus signals produced by SA receptors. For tactile sensing, stretchable triboelectric interlinked films (TIFs) are employed. These films use innovative triboelectric materials with differential electron affinities and operate based on the TENG‐based sensing mechanism, generating intermittent pulsed signals that emulate the FA receptor responses. This mechanism forms a triboelectric artificial synapse, enabling the digital encoding of tactile information and enhancing the adaptability of the E‐armor in the complex intestinal environment. An autonomous compliance control system is developed to interpret tactile and strain sensing signals, enabling the robot to sense its surrounding environment and guide continuous bending and posture adjustments. This system facilitates optimized intracolonic navigation by performing dynamic postural adjustments based on bimodal sensory feedback. In addition, a digital twin‐based human–robot interface is implemented to dynamically replicate the robot's contact states and bending configurations in real time, significantly improving colonoscopic visualization (Figure 1b‐i).

This article is organized as follows: Section 2 presents the bioinspired design and working principles of the stretchable E‐armor, detailing its 3D crosslinked structure, triboelectric artificial synapse mechanism, and hydrogel‐based strain sensing. Section 3 discusses the experimental outcomes, including the tactile recognition framework (featuring a digital encoding strategy combined with CNN‐LSTM networks), strain sensing characterization during robot bending, compliance control based on bimodal sensory feedback, and digital twin‐based visualization for robot navigation. Section 4 summarizes the key findings and their potential clinical implications. Section 5 outlines the materials and fabrication methods used in this study.

## 2 Design and Working Mechanism

The 3D structured stretchable E‐armor for colonoscopic continuum robot achieves a long‐distance coverage of 300 mm, including 48 tactile sensing points and 12 strain sensing edges nested onto the robot's discs, which allows simultaneous and multiplexed sensing of tactile and strain modalities. The E‐armor can be divided into three sub‐units and each E‐armor unit incorporates two primary components: sensing belts and sensing rings. For each unit, four sensing belts and four sensing rings are attached to the continuum robot through four supporting rings, forming a crosslinked sensing‐net structure (Figure 1b‐ii).

The sensing belt consists of a flexible substrate made of Ecoflex, a hydrogel sensitive element for strain sensing (S‐hydrogel element), and an isolation film for encapsulation and separation (Figure S1a‐i,b, Supporting Information). The S‐hydrogel element is a double‐network structure hydrogel, formed by the crosslinking of sodium alginate (SA) and polyacrylamide (PAM), with sodium chloride (NaCl) added to improve ionic conductivity. Within the hydrogels, sodium ions (Na⁺) and chloride ions (Cl⁻) not only trigger supramolecular assembly but also significantly enhance conductivity, rendering the hydrogel an ideal ionic conductor for stretchable electronics.^[^ ^73^, ^74^ ^]^ The hydrogel's schematic structure, molecular formula, transparency, stretchability, and SEM images are shown in Figure S2, Supporting Information. The tactile sensing ring comprises a tactile hydrogel (T‐hydrogel) electrode, inner and outer triboelectric interlinked films (TIFs), and an aluminum (Al) electrode for signal transmission (Figure S1a‐ii,b, Supporting Information). In the TIF design, particles of polyvinyl alcohol (PVA), polytetrafluoroethylene (PTFE), and polyamide (PA) are each doped into Ecoflex, to fabricate PVA‐Ecoflex, PTFE‐Ecoflex, and PA‐Ecoflex composites. PA, a tribopositive material, and PTFE, a tribonegative material, modify the inherent triboelectric properties of Ecoflex. Hence, the inner and outer TIFs, comprising PA‐Ecoflex and PTFE‐Ecoflex, respectively, exhibit distinct triboelectric properties, enabling the generation of specific tactile signals through triboelectric interaction. The triboelectrically neutral PVA maintains its neutrality when blended with Ecoflex. Thus, PVA‐Ecoflex is used as a transition layer between PA‐Ecoflex and PTFE‐Ecoflex, effectively preventing charge drift between the triboelectric surfaces. SEM images of these materials are provided in Figure S1d, Supporting Information. The physical appearance of the continuum robot covered with the E‐armor shows its excellent conformity to the robot's multi‐DOF bending structure (Figure S1c, Supporting Information). The feasability of using the E‐armor in intestinal environments is validated by performing biocompatibility evaluations on all flexible materials, including the SA/PAM/NaCl conductive hydrogel, PA‐Ecoflex, PVA‐Ecoflex, PTFE‐Ecoflex, and pure Ecoflex. Using both Cell Counting Kit‐8 (CCK‐8) assays and live/dead fluorescence staining, all materials are confirmed to exhibit excellent biocompatibility (Text S1 and Figures S3 and S4, Supporting Information).

The application scenario and perception strategy for the E‐armor's strain sensing belts are illustrated in Figure 1c. In each sensing belt, the hydrogel serves as the strain sensing element.^[^ ^75^ ^]^ When the sensing belt is stretched or compressed, the cross‐sectional area of the hydrogel changes, altering its conductivity and consequently changing its resistance. This enables the conversion of mechanical strain into an electrical resistance signal. Each E‐armor unit contains four sensing belts arranged to form a spatial strain sensing net (Figure 1c‐i). The incorporation of Na^+^ and Cl^−^ ions significantly enhances the hydrogel's conductivity; while, its double‐network structure improves flexibility, enabling the belts to conform to the robot's multi‐joint bending motions (Figure 1c‐ii). As the continuum robot bends within the intestinal environment, the four sensing belts experience strain in varying magnitudes and directions. These differences result in distinct resistance changes in each belt, which are then used to infer both the bending angle and direction of the continuum robot (Figure 1c‐iii).

The application scenario and perception strategy for the tactile sensing rings of the E‐armor are described in Figure 1d. Each sensing ring is equipped with 4 tactile sensing points, resulting in 16 tactile sensing points per E‐armor unit, which together form a tactile sensing net (Figure 1d‐i). Each tactile sensing point comprises a pair of triboelectric layers: hemispherical PTFE‐Ecoflex (or PA‐Ecoflex) surface and a flat PA‐Ecoflex (or PTFE‐Ecoflex) surface, separated by a 0.5 mm air gap, collectively defined as a TENG pair. The outer triboelectric surface is hemispherical; while, the inner surface is flat. When mechanical contact occurs, the TENG pair converts contact energy into electrical signals via the combined effects of electrostatic induction and the triboelectric effect (Figure 1d‐ii). The signal output of this triboelectric artificial synapse resembles the pulsed stimulus signals generated by FA receptors in biological skin. This behavior arises from the opposite triboelectric polarities of PA‐Ecoflex and PTFE‐Ecoflex. The electrode beneath the PTFE‐Ecoflex layer generates a signal waveform characterized by a peak followed by a valley, denoted as “1.” In contrast, the electrode beneath the PA‐Ecoflex layer produces a waveform with a valley followed by a peak, denoted as “−1”. If the electrode does not generate a signal, it is encoded as “0” (Figure 1d‐iii). This strategy, digitally encoding triboelectric signals based on their waveform characteristics, forms the foundation for the signal digitization method used in the E‐armor.

During operation, tactile signals generated by contact between the continuum robot and the intestinal wall trigger bending actions. When a specific position is touched, each E‐armor unit generates eight channel signals with different characteristics. These triboelectric artificial synaptic signals are digitally encoded as “1”, “−1,” and “0,” forming a tactile sensing sequence represented as a 1 × 8 matrix. This matrix is used for both tactile point position recognition and robot posture adjustment. The contact location on the E‐armor is accurately identified using a convolutional neural network and long short‐term memory (CNN‐LSTM) framework. The CNN component extracts spatial features from the signal matrix, whereas the LSTM component captures temporal dependencies in the sequence, enabling robust and precise interpretation of tactile events. This hybrid approach leverages the strengths of CNNs in recognizing spatial patterns and those of LSTMs in capturing temporal dependencies, enabling precise identification of contact locations on the E‐armor. Simultaneously, strain signals are recorded in real time to inform posture adjustments as these signals reflect the bending angles of the robot's joints; and thus, enable accurate posture recognition. By continuously collecting and analyzing both tactile and strain signals, the system dynamically adjusts the posture of the robot, enabling real‐time, autonomous navigation within the intestinal environment. In addition, strain data from the robot's joints are integrated into a digital twin interface, which reconstructs a 3D animation of the robot's posture in regions of the intestine that are not directly visible. This visualization provides operators with real‐time feedback on the robot's internal positioning, enhancing situational awareness and improving operational precision. By offering a clear representation of the robot's movements and orientation, the digital twin significantly improves the efficiency and accuracy of the procedure. This autonomous navigation strategy functions as a closed‐loop feedback system, continuously linking tactile and strain information to guide the robot's behavior (Figure 1e). Three E‐armor units were integrated into the continuum robot using a prestretched approach, enabling the sensing belts to achieve bidirectional strain sensing capabilities. The supporting rings and the robot's discs were bonded with adhesive to ensure that the tactile sensing points on the E‐armor remained fixed relative to the robot's surface, even during structural deformation and stretching. Detailed descriptions of the robot's design, structure, and control system are provided in Texts S2 and S3, and Figures S5 and S6, Supporting Information.

## 3 Results and Discussion

### 3.1 Digital Encoding Strategy of E‐Armor Tactile Sensing Signal

**Figure 2:**
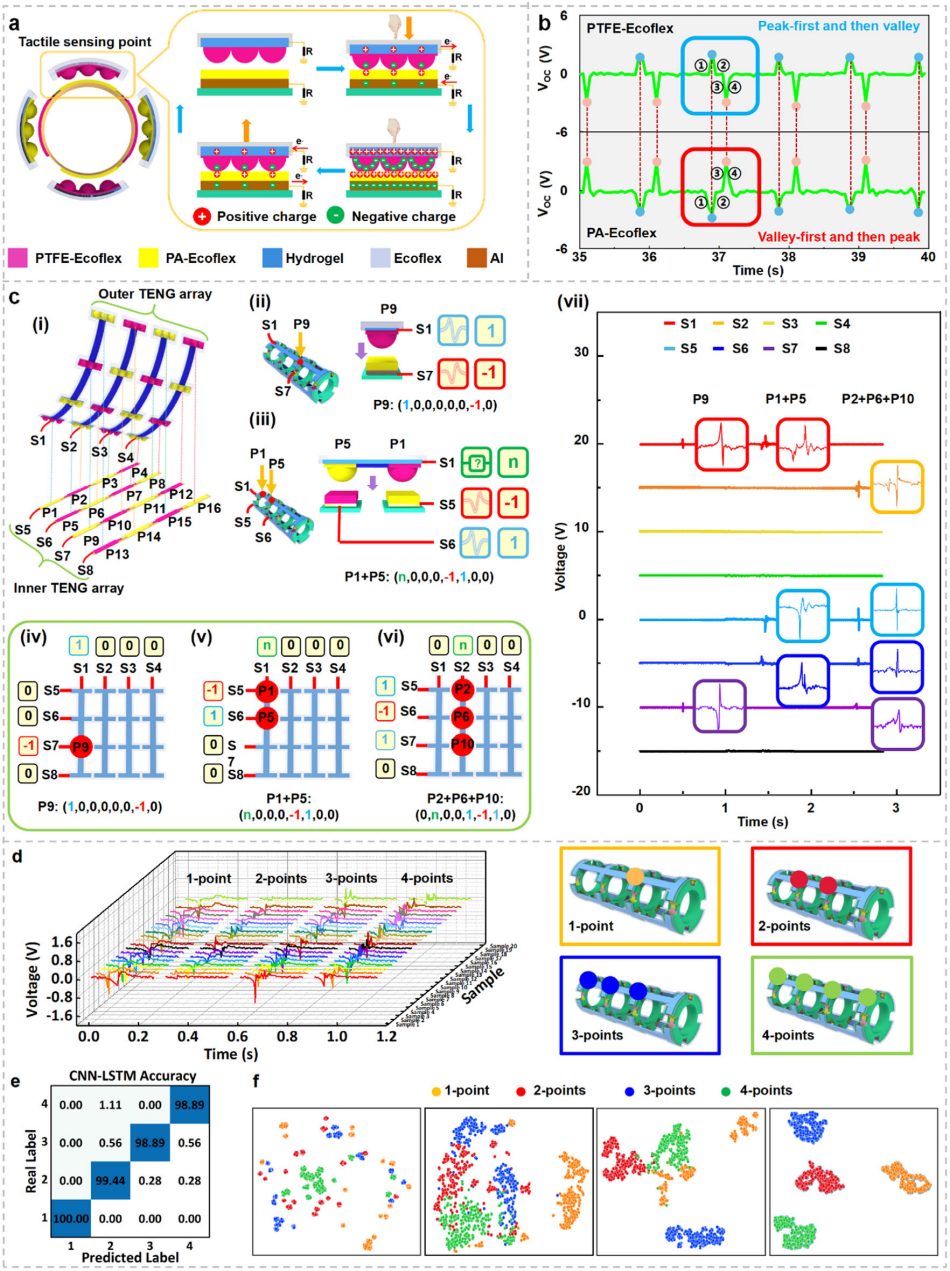
Digital encoding strategy of E‐armor tactile sensing signals. a) Working mechanisms of tactile sensing. b) TENG signal waveforms generated by PTFE‐Ecoflex and PA‐Ecoflex. c) Digital encoding strategy of E‐armor unit tactile sensing signal. c‐i) Naming convention for tactile sensing points. c‐ii) Digital encoding strategy for a single‐point contact. c‐iii) Digital encoding strategy of multi‐point contact. c‐iv) Digital encoding of tactile signals exemplified by P9. c‐v) Digital encoding of tactile signals exemplified by simultaneous contact with P1 and P5. c‐vi) Digital encoding of tactile signals exemplified by simultaneous contact with P2, P6, and P10. c‐vii) Real‐time voltage signal waveforms corresponding to examples (c‐iv–vi). d) Real‐time voltage waveforms of the TENG tactile signal under four cases of contact. e) Confusion matrices showing classification accuracy using the CNN‐LSTM model. f) 2D point cloud visualization using t‐SNE for signal classification.

When the continuum robot contacts the intestinal wall, the tactile sensing rings generate signals at each tactile sensing point. The working principle of this mechanism is illustrated in **Figure** **2**a. Upon compression of a tactile sensing point, the PTFE‐Ecoflex and PA‐Ecoflex films come into contact. Owing to their differing triboelectric polarities, electron transfer occurs: free electrons flow from the PTFE‐Ecoflex hydrogel electrode to ground and from ground to the Al electrode beneath the PA‐Ecoflex layer (Phases 1 and 2). When the object retracts, the electron flow reverses direction (Phases 3 and 4). The hemispherical structure of the tactile sensing surface ensures optimal contact and triboelectric interaction, minimizing surface charge drift. The opposite tribonegativities of PA‐Ecoflex and PTFE‐Ecoflex leads to the production of paired voltage signals with distinct polarities. Specifically, the electrode beneath PTFE‐Ecoflex produces peak‐first‐then‐valley waveform, denoted as “1,” whereas the electrode under PA‐Ecoflex produces a valley‐first‐then‐peak waveform, denoted as “−1” (Figure 2b). A lack of signal from an electrode is encoded as “0.” This study comprehensively evaluates the performance, stability, and sensitivity of each TENG pair under various conditions. These include material preparation and optimization, testing with different external contact materials, analysis of strain effects, pressure variation testing, contact frequency testing, signal waveform analysis, sensitivity calibration, and repeatability assessment (Text S4 and Figure S7, Supporting Information). Given the potential application of E‐armor in the intestinal environment, the possible influence of the presence of polyps or tumors on its performance must be assessed. As polyps and tumors exhibit distinct stiffness levels depending on their pathological conditions, the tactile sensing signals generated by materials of different stiffness were systematically investigated (Text S5 and Figure S8, Supporting Information). In addition, the intestinal environment presents a wide range of pH conditions. To ensure robustness, the stability of the tactile sensing capabilities of the E‐armor was evaluated under varying pH levels (Text S6 and Figure S9, Supporting Information).

The arrangement and naming convention of the tactile sensing points are clearly illustrated by fully disassembling the E‐armor unit and presenting a magnified view (Figure 2c‐i). When all sensing belts and rings are laid flat, the E‐armor unit reveals a bilayer array structure comprising an outer TENG array and an inner TENG array. The outer TENG array comprises four outer TIFs and their corresponding T‐hydrogel electrodes. These T‐hydrogel electrodes function as co‐electrodes, responsible for transmitting triboelectric signals from the four tactile sensing points on each outer TIF. Similarly, the inner TENG array includes four inner TIFs and their respective Al electrodes, which also act as co‐electrodes to transmit TENG signals from the four tactile sensing points on each inner TIF. Each of the four outer TIFs is connected vertically to a dedicated signal channel, labeled S1–S4, whereas each of the four inner TIFs is connected horizontally to its own signal channel, named S5–S8. All outer and inner TIFs are crosslinked to form 16 tactile sensing points, labeled P1–P16. Each sensing point is located at the intersection of one outer and one inner TIF. At each intersection, the triboelectric materials from the outer and inner TIFs are selected to have opposite triboelectric polarities, ensuring effective signal generation upon contact. When a tactile point is activated by contact, a TENG signal pair is generated and transmitted via the corresponding outer and inner TIF signal channels. The location and encoded signal of each tactile sensing point are expressed as Pi (S1, S2, S3, S4, S5, S6, S7 and S8). This enables a digitized encoding for identifying tactile events. Consider the example of P9 (Figure 2c‐ii)), located at the orthogonal intersection of the outer TIF associated with S1 and the inner TIF associated with S7. In this case, the upper triboelectric layer is PTFE‐Ecoflex, and the bottom layer is PA‐Ecoflex. Upon contact, the signal from S1 exhibits a peak‐valley waveform, encoded as 1. The signal from S7 shows a valley‐peak waveform, encoded as −1 (Figure 2c–iv). All other channels (S2–S6,S8) show no signal and are encoded as 0. Thus, the tactile signal for P9 is encoded as (1, 0, 0, 0, 0, 0, −1, 0). The digital sequence uniquely identifies the contact point and enables effective mapping of tactile input. Additional examples, such as contact events at P6 and P15 are illustrated in Text S7 and Figure S10a,b, Supporting Information.

The actual operating environment of a continuum robot within the intestine is highly complex, often involving frequent multipoint contact events. A key challenge in designing common‐electrode (co‐electrode) triboelectric devices lies in the ability to recognize individual signals and mitigate interference in shared signal channels (co‐channels). To address this, the digital encoding strategy proposed in this study not only enables accurate identification of single‐point contact events but also effectively handles multi‐point contacts. The encoding mechanism for multi‐point tactile events in the E‐armor is shown in Figure 2c‐ii. Take the example of simultaneous contacts at tactile sensing points P1 and P5. P1 and P5 are both located along the same outer TIF channel S1, but different inner TIF channels S5 and S6, respectively. The signal transmitted by channel S1 is fundamentally a coupled signal, whereas the signals conveyed by channels S5 and S6 are essentially simple signals. It is clear that the simple signals generated on inner TIF channels S5 and S6 result from the separated contact of PA‐Ecoflex and PTFE‐Ecoflex, forming a valley‐peak waveform coded as −1 and a peak‐valley waveform coded as 1, respectively. The signal on outer TIF channel S1 is a coupled signal generated by the respective contacts of PA‐Ecoflex and PTFE‐Ecoflex, coded as “n”. Theoretically, its value should be the coupling of the inverse signals of inner TIF channels S5 and S6. Therefore, the tactile sequence for P1 + P5 can be coded as (*n*, 0, 0, 0, −1, 1, 0, 0) (Figure 2c‐v). The encoding principle remains the same even in complex scenarios, such as when three tactile sensing points share a common outer or inner TIF. For instance, when P2, P6, and P10 are contacted simultaneously (Figure 2c‐vi), all of which share outer TIF channel S2, the signal on S2 becomes a coupled waveform, denoted as “*n*.” Meanwhile, their respective inner TIF channels S5–S7 are in contact with the materials PTFE‐Ecoflex, PA‐Ecoflex, and PTFE‐Ecoflex, respectively; hence, these simple signals are coded as 1, −1, and 1, respectively. As a result, the tactile sequence code for the event P2+P6+P10 is (0, *n*, 0, 0, 1, −1, 1, 0). The corresponding real‐time voltage waveforms for these three scenarios are shown in Figure 2c‐vii, with enlarged views illustrating the waveform characteristics of each contact point. As expected, all signals demonstrate the appropriate sequence of peaks and valleys. Notably, as the number of contact points increases, the complexity of the coupled signals also rises. This digital encoding strategy remains effective even in cases of four‐point simultaneous contact (Figure S10c, Supporting Information). Overall, it enables robust identification of both single‐point and multi‐point contact events, even under conditions of signal interference along shared channels (Text S8 and Figure S11, Supporting Information).

As discussed earlier, coupled tactile signals are formed by the inverse superposition of individual simple signals orthogonal to the contact channel. This renders the pattern recognition of such coupled signals, particularly under arbitrary multipoint contact combinations, highly challenging. The real‐time voltage waveforms for 1‐point, 2‐points, 3‐points, and 4‐points contacts on a co‐electrode are shown in Figure 2d. As illustrated, coupled tactile signals often appear weakened or exhibit highly complex waveforms (Figure S12, Supporting Information), rendering accurate pattern recognition challenging. To overcome this, a CNN‐LSTM neural network framework enhanced with a self‐attention mechanism is developed (Figure S13a, Supporting Information). The framework operates in two key stages: the CNN module extracts local spatial features from the collected signal‐strength sequences, whereas the LSTM module, equipped with a self‐attention mechanism, captures long‐term dependencies within these sequences to enhance convergence speed and recognition performance. The attention mechanism calculates similarities between elements at different positions within the signal sequence, enabling the model to understand inter‐element relationships and maintain high accuracy when predicting long or complex sequences. This mechanism also prioritizes informative features by assigning higher weights to key patterns; while, suppressing redundant or noisy data, thereby improving robustness. With this architecture, the proposed CNN‐LSTM method achieves an average recognition accuracy of 99.31% across varying contact scenarios (Figure 2e), demonstrating its effectiveness in decoding complex, coupled tactile signals.

The superior performance of the proposed CNN‐LSTM model was validated by comparing its recognition accuracy with that of conventional machine learning algorithms, including CNN, LSTM, and support vector machine (SVM). The results clearly demonstrated that the CNN‐LSTM architecture outperformed all three, achieving higher recognition accuracy for multipoint tactile signal classification (Text S9 and Figure S13b, Supporting Information). In addition, the computational efficiency and feasibility for real‐time processing of CNN‐LSTM were compared with those of other methods, further highlighting its advantages in low‐latency applications (Text S10, Supporting Information). For an intuitive understanding of the classification process, t‐distributed stochastic neighbor embedding (t‐SNE) was used to visualize the model's internal feature transformations at different network layers (Figure 2f). In the early layers, the point clouds representing different contact categories showed significant overlap. However, as the network deepened, these point clouds became clearly separable, illustrating the model's ability to extract distinct and discriminative features through its hierarchical structure. The specific network structure and parameters of the CNN‐LSTM model are listed in Table S4, Supporting Information. Further, a comparative analysis was conducted between the E‐armor and other TENG‐based sensor arrays employing co‐electrode configurations. The soft, stretchable design of the E‐armor, combined with its ability to perform multipoint recognition and eliminate signal crosstalk, significantly enhances its applicability for continuum robots operating in the constrained and complex environment of the intestine (Table S10, Supporting Information).

### 3.2 Strain Sensing Characterization of the E‐Armor

**Figure 3:**
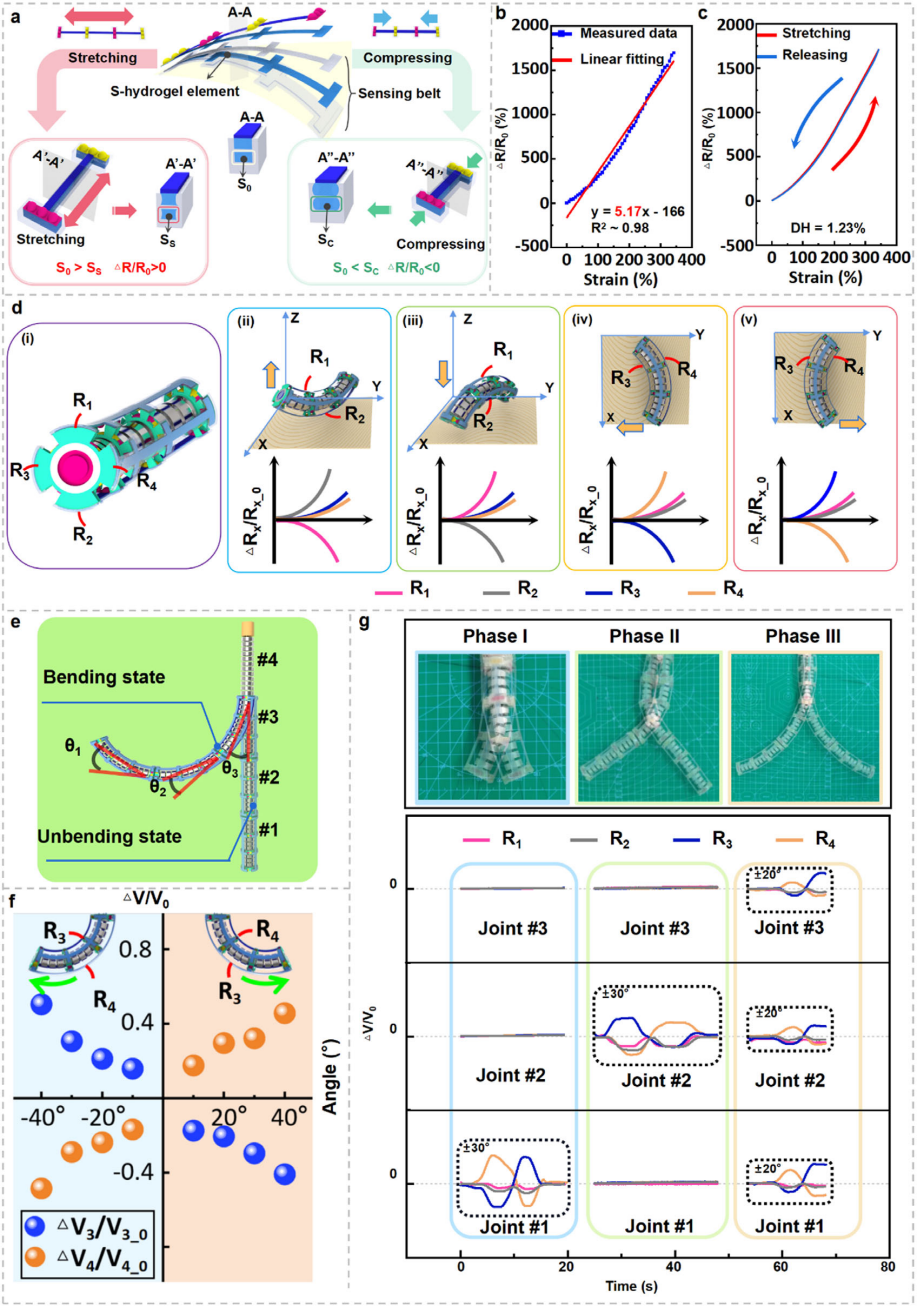
Strain sensing performance and continuum robot posture recognition strategy of the E‐armor. a) Working mechanisms of strain sensing. b) Relationship between the relative resistance change rate and strain of the sensing belt. c) Hysteresis of the sensing belt. d) Continuum robot bending posture recognition strategy for the joint of the E‐armor unit. d‐i) Naming of the four sensing belts on the E‐armor unit. d‐ii–v) Relationship between the bending direction of continuum robot joint and the relative resistance change rates. e) Naming convention and schematic of bending angles for E‐armor units on continuum robot. f) Relationship between the relative voltage change rate of the E‐armor unit and the bending angle of continuum robot single joint. g) Posture recognition of single‐joint and multi‐joint configurations in continuum robots using the E‐armor.

In addition to tactile perception, the colonoscopic continuum robot must detect its own deformations via the strain sensing belts of each E‐armor unit. When the robot undergoes tensile deformation, the cross‐sectional area of the S‐hydrogel element within the sensing belt reduces from its initial state (*S*_0_) to a smaller area (*A’*–*A’* denoted as (*S*_S_)). Assuming a constant volume of the S‐hydrogel, this decrease in cross‐sectional area, accompanied by an increase in length, leads to a rise in resistance, thereby amplifying the relative resistance change rate. Conversely, during compressive deformation, the cross‐sectional area of the S‐hydrogel element increases from *S*_0_ to *A″*–*A″* denoted as (*S*_C_); while, its length shortens. This change decreases the resistance of the element, resulting in a reduced relative resistance change rate (**Figure** **3**a; Text S11, Supporting Information). The resistive sensing characteristics of sensing belts fabricated using materials such as Ecoflex, PA‐Ecoflex, PTFE‐Ecoflex, PVA‐Ecoflex, and SA/PAM/NaCl conductive hydrogels are systematically evaluated. The relationship between the relative resistance change rate (*ΔR/R*_0_) and strain across a stretching range of 0–300% is illustrated in Figure 3b. It is found that the relative resistance change rate and strain exhibit an approximate linear relationship, with a linear fitting of *y* = 0.05*x* – 1.66. Hence, the sensitivity of the sensing belt is determined as 5.17. An excellent linear fit between *ΔR/R*_0_ and strain can be observed, with a high *R^2^* of 0.98. The hysteresis loop of the sensing belt during stretching and releasing within a strain range of 300% is shown in Figure 3c. The sensing belt exhibits a minor hysteresis characteristic of only 1.23%. Given that each E‐armor unit integrates tactile sensing rings and strain sensing belts, their potential interaction is assessed. The sensing belts are stretched to strains of 50%, 100%, and 150% using a linear motor, after which, tactile events are applied at a frequency of 0.3 Hz and pressure of 1 kPa. The results show that the relative resistance change rate of the sensing belt remains stable during each tactile event. In addition, the repeatability of the strain sensing belts is evaluated under dynamic loading for 1000 s. The relative resistance change rate remains stable with negligible offset (Figure S14, Supporting Information). The environmental robustness is further assessed by testing and characterizing the strain sensing capabilities of the E‐armor strains across varying pH environments to evaluate their feasibility for intestinal applications (Text S12 and Figure S15, Supporting Information).

Each joint of the continuum robot is equipped with one E‐armor unit, comprising four strain sensing belts evenly distributed around the joint at 90° intervals. During joint bending, the relative resistance change rates of these sensing belts differ based on their positional deformation, which enables inference of both the bending angle and direction. The resistances of the four sensing belts are designated as *R*_1_, *R*_2_, *R*_3_, and *R*_4_ (Figure 3d‐i). In a representative scenario where the robot joint bends upward, the upper sensing belt undergoes compression, resulting in a decrease in its corresponding resistance *R*_1_. Conversely, the lower sensing belt experiences stretching, leading to an increase in *R*_2_. The lateral sensing belts (right and left) undergo slight stretching, causing modest increases in *R*_3_ and *R*_4_, respectively. These resistance variations are expressed as relative resistance change rates, symbolized by *ΔR_x_/R_x_0_*, where *ΔR_x_* represents the real‐time resistance change value and *R_x_0_* stands for the initial resistance value. Notably, *ΔR*_1_ */R*_1_ *___*_0_ manifests a pronounced decrease and *ΔR*_2_ */R*_2_ *___*_0_ exhibits a considerable increment, whereas *ΔR*_3_ */R*_3_ *___*_0_ and *ΔR*_4_ */R*_4_ *___*_0_ display minor increments (Figure 3d‐ii). The bending upward scenario can be assessed based on *ΔR_x_/R_x__*_0_. In addition, minor stretching is observed on the side‐sensing belts, resulting in a slight increase in the resistances *R*_3_ and *R*_4_. Hence, the bending downward scenario can also be evaluated based on *ΔR_x_/R_x__*_0_ (Figure 3d‐iii). The conditions corresponding to the leftward and rightward bending of the robot joint are depicted in Figures 3d‐iv and 3d‐v, respectively. The bending angle and direction of the robot joint can be determined based on the relative resistance change rate values in the different bending states of *R*_1_ to *R*_4_. This provides valuable information for the comprehensive assessment of the robot's posture.

The continuum robot used in this study comprises four joints, sequentially named from the distal end to the base as “Joint #1” to “Joint #4” (Figure 3e). The first three joints (Joints #1–#3) are each covered with an E‐armor unit. Each of these joints possesses two degrees of freedom (2‐DOF), providing the continuum robot with 6‐DOF and enabling spatial motion with superimposed bending angles of *θ*_1_, *θ*_2_, and *θ*_3_. Joint #4 serves primarily as a support and extension structure for the preceding three joints. When a robot joint bends leftward and rightward at different angles, the calibrated relative voltage change rates of the sensing belts of the E‐armor unit are symbolized by *ΔV_x_/V_x__*_0_ (Figure 3f). Here, *ΔV_x_* represents the real‐time voltage change value and *V_x__*_0_ stands for the initial voltage value. The relative voltage change rates of the sensing belts with resistances *R*_3_ and *R*_4_ are obtained through the signal amplifier circuit (Text S13 and Figure S16, Supporting Information) and are denoted as *ΔV*_3_ */V*_3_ *___*_0_ and *ΔV*_4_ */V*_4_ *___*_0_. When a joint is bent to the left from 0° to −40°, the corresponding relative voltage change rates for *R*_3_ and *R*_4_ at each angle can be obtained. Specifically, the relative voltage change rate of *R*_3_, which experiences compression during leftward bending, shows an increasing trend; while, that of *R*_4_, subjected to stretching, exhibits a decreasing trend. Conversely, when the robot joint bends rightward from 0° to 40°, the relative voltage change rates of *R*_3_ and *R*_4_ exhibit opposite trends. The bending angle of each joint is individually calibrated and calculated based on these voltage changes. Consequently, the total bending angle of the continuum robot is determined by summing the bending angles of its individual joints.

The real‐time signals of the relative voltage change rates of the four sensing belts under both single‐ joint and multiple‐joint bending conditions are shown in Figure 3g. In phase I, Joint #1 was actuated to swing 30° to the right and then to the left. During this motion, the relative voltage change rates of sensing belts *R*_3_ and *R*_4_ exhibited distinct alternating increases and decreases; while, *R*_1_ and *R*_2_ only showed minor variations. This is because, during the swing process, *R*_3_ experienced stretching, followed by compression, whereas *R*_4_ experienced the opposite trend, and *R*_1_ and *R*_2_ underwent only small stretches. In Phase II, similar 30° swings to the left and right were applied to Joint #2. Correspondingly, during this process, the relative voltage change rate of *R*_3_ experienced compression followed by stretching, whereas the relative voltage change rate of *R*_4_ experienced the opposite strain. The relative voltage change rates at *R*_1_ and *R*_2_ maintained a consistent decreasing trend. In phase III, simultaneous rightward and leftward swings of 20° were applied to Joints #1, #2, and #3. Evidently, from the graphs, each joint displayed a trend where the relative voltage change rate of *R*_3_ first decreased and then increased, and the relative voltage change rate of *R*_4_ first increased and then decreased. In contrast, *R*_1_ and *R*_2_ showed negligible variations in their voltage change rates. The scale in the background panel confirmed a total bending angle of 60° for the continuum robot, confirming the feasibility of the joint bending‐angle superposition strategy. The posture recognition of single‐joint and multi‐joint configurations in continuum robot using the E‐armor was filmed, demonstrating its effectiveness (Movie S1, Supporting Information). Further, the softness of the continuum robot integrated with the E‐armor, critical for ensuring human–robot interaction safety in practical applications, was thoroughly elaborated and discussed (Text S14, Supporting Information).

### 3.3 Compliance Control Strategy of Continuum Robot Based on E‐Armor Sensing Signals

**Figure 4:**
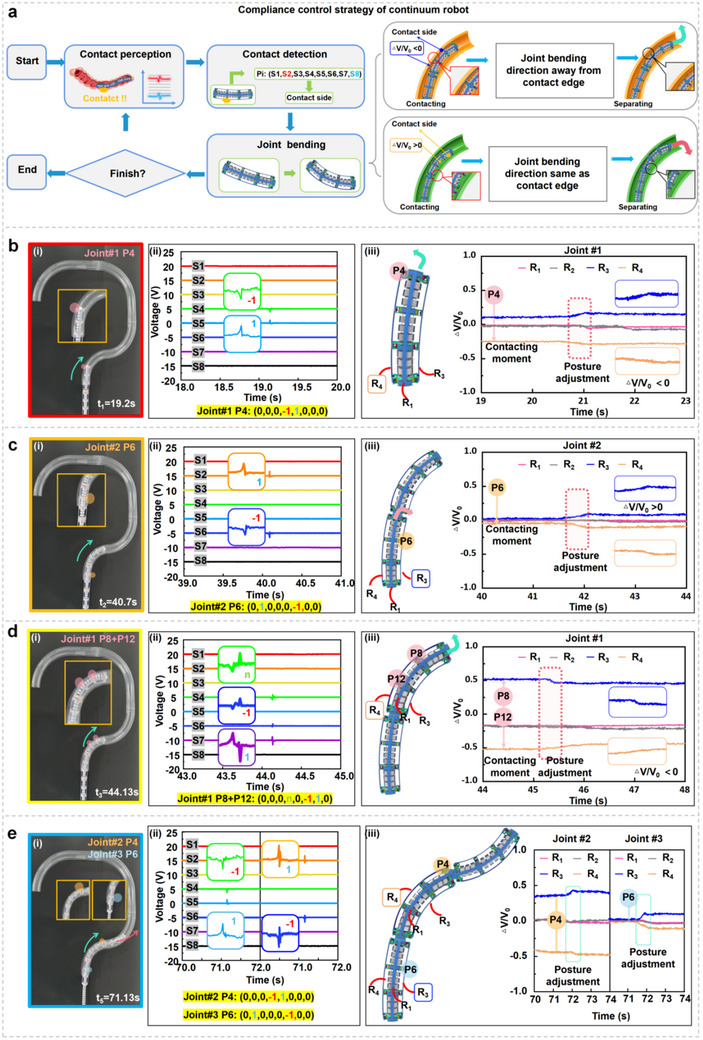
Compliance control strategy and autonomous navigation of a continuum robot integrated with E‐armor in an intestine‐shaped acrylic pipeline. a) Compliance control strategy of continuum robot. b) Demonstration of the compliance control of single‐point on Joint #1. b‐i) Screenshot of the contact point P4 on Joint #1. b‐ii) Tactile signals of the contact point P4 on Joint #1. b‐iii) Strain signals of the contact point P4 on Joint #1. c) Demonstration of the compliance control of single‐point on Joint #2. c‐i) Screenshot of the contact point P6 on Joint #2. c‐ii) Tactile signals of the contact point P6 on Joint #2. c‐iii) Strain signals of the contact point P6 on Joint #2. d) Demonstration of the compliance control of multi‐point on Joint #1. d‐i) Screenshot of the contact points P8 and P12 on Joint #1. d‐ii) Tactile signals of the contact points P8 and P12 on Joint #1. d‐iii) Strain signals of the contact points P8 and P12 on Joint #1. e) Demonstration of the compliance control of Joint #2 and Joint #3. e‐i) Screenshot of the contact points P4 on Joint #2 and P6 on Joint #3. e‐ii) Tactile sensing signals of the contact points P4 on Joint #2 and P6 on Joint #3. e‐iii) Strain sensing signals of the contact points P4 on Joint #2 and P6 on Joint #3.

To safely interact with the intestinal environment, continuum robots require excellent sensing capabilities and compliance control strategies. In this study, when a continuum robot enters the intestine and makes contact with a tactile sensing point, the E‐armor generates a triboelectric tactile sequence code. The system identifies the coordinates of the tactile sensing point and controls the corresponding joint to apply a deflection aligned with the tactile force direction. To optimize the navigation of continuum robot in confined environments while enabling it to achieve stable advancement with the minimal number of adjustments, we implement an optimized strategy that leverages the unique bimodal sensing capability of the E‐armor. The improved system now monitors the relative voltage change rate (*ΔV_x_/V_x_0_*) upon contact detection. A negative ratio (<0) maintains the bending away from the contact surfaces, whereas a positive ratio (>0) aligns the motion with the contact forces. This adaptive approach, illustrated in **Figure** **4**a, effectively prevents intestinal oscillations by dynamically adjusting the movement based on the correlated tactile‐strain feedback. Unlike previous methods that rely on single‐modal inputs or preset thresholds,^[^ ^56^, ^60^, ^62^, ^63^, ^76^, ^77^ ^]^ our solution enables safer navigation through deformable biological environments by intrinsically combining both sensing modalities. A detailed comparison with existing flexible sensor‐assisted strategies for continuum robots is provided in Table S11, Supporting Information. To validate the compliance control strategy, Joint #1 of the continuum robot covered with the E‐armor unit is programmed to realize autonomous navigation through pipelines with three bending angles: 90°, 110°, and 130°. The operational results demonstrate the effectiveness of this strategy (Text S15, Figure S17, and Movie S2, Supporting Information).

Considering the complexity of the intestinal environment, multiple joints must collaboratively bend and move forward without excessive pressure on the intestinal wall. During this process, the posterior joint may affect the position of the adjusted anterior joint, leading to further collision with the intestinal wall. To ensure smooth and precise crawling, a compliance control strategy for alleviating joint coupling was introduced (Text S16 and Text S17, Supporting Information). In this study, an acrylic pipeline structure resembling the intestine was constructed, and an autonomous navigation test of a continuum robot was conducted. Three E‐armor units integrated into the robot joints were used to capture the robot's tactile and strain information throughout the crawling process. During the automatic navigation process of the continuum robot, four representative moments describing the compliance control strategy were captured. The continuum robot advanced through an acrylic pipeline at a speed of 3.5 mm s^−1^. During the navigation process, Joint #1 first entered the pipeline and the tactile signals of channels S1–S8 were recorded. At 14.2 s, Joint #1 contacted the wall of the pipeline at P4 point (Figure 4b‐i). The tactile signal waveform of S4 channel was peak‐first and then valley, recorded as “ −1;” while, that of S5 channel was valley‐first and then peak, recorded as “1.” Therefore, the tactile sequence code for P4 was (0,0,0, −1,1,0,0,0) (Figure 4b‐ii). As P4 was located on the sensing belt *R*_4_, at this moment, *∆V*_4_ */V*_4_ *___*_0_ < 0. According to the compliance control strategy, the direction of joint bending should move away from the contact edge. Consequently, the system provides a command for posture adjustment of the continuum robot. *ΔV*_4_ */V*_4_ *___*_0_ decreased again, indicating that Joint #1 continued to bend away from the contact edge (Figure 4b‐iii). Joint #2 began to engage with the pipeline when Joint #1 of the continuum robot entered the pipeline completely. At 40.7 s, Joint #2 was in contact with the pipeline at P6 (Figure 4c‐i). By analyzing and digitizing the tactile signal waveforms collected from channels S1–S8, the tactile sequence code for P6 was obtained as (0,1,0,0,0,−1,0,0) (Figure 4c‐ii), indicating that P6 was positioned on the sensing belt *R*_3_. *ΔV*_3_ */V*_3_ *___*_0_ > 0. According to the compliance control strategy, the system issued a posture adjustment command to Joint #2, causing it to bend in the same direction as the contact edge (Figure 4c‐iii). The continuum robot continued to move into the pipeline. At 44.1 s, the system detected a multipoint tactile event at points P8 and P12 of Joint #1 (Figure 4d‐i). They collectively utilized the common S4 channel, resulting in a coupled signal output of S4, denoted as “*n*”. Therefore, the tactile sequence code generated by the simultaneous contacts of P8 and P12 was (0,0,0,*n*,0,−1,1,0) (Figure 4d‐ii). Accordingly, P8 and P12 were identified to be located on sensing belt *R*_4_. At this moment, *ΔV*_4_ */V*_4_ *___*_0_ < 0 and the system issued a command for Joint #1 to continue bending away from the contact edge. After Joint #2 of the continuum robot was fully engaged in the pipeline, Joint #3 began to enter the pipeline (Figure 4d‐iii). At 71.1 s, the identified contact points were P4 on Joint #2 and P6 on Joint #3 (Figure 4e‐i–ii)). By analyzing the relative voltage change rates of the sensing belts where these two points were located, it was found that *ΔV*_4_ */V*_4_ *___*_0_ < 0 on Joint #2 and *ΔV*_3_ */V*_3_ *___*_0_ > 0 on Joint #3, respectively. Consequently, the system applied different bending commands to these two joints; Joint #2 was instructed to bend away from the contact edge, whereas Joint #3 was instructed to bend closer to the contact edge (Figure 4e‐iii). The autonomous navigation of a continuum robot integrated with an E‐armor in an intestine‐shaped pipeline was thus demonstrated (Movie S3, Supporting Information). The complete tactile signals, strain signals, and tactile sequence codes were collected and recorded (Text S19 and Figure S23, Supporting Information). Only a few continuum robots integrated with E‐skin have achieved the incorporation and system feedback of sensory information. In this work, the tactile sensing information of the E‐armor was fed back to the system for identification of multipoint contact events and adjustment of the robot's posture accordingly. In addition, the stability of the compliance control strategy was thoroughly analyzed (Text S18, Supporting Information). This enabled the continuum robot to adapt to complex multicurved intestinal structures and achieve adaptive navigation within the intestine (Table S9, Supporting Information).

### 3.4 Digital‐Twin‐Based 3D Posture Visualization Interface of Continuum Robot

**Figure 5:**
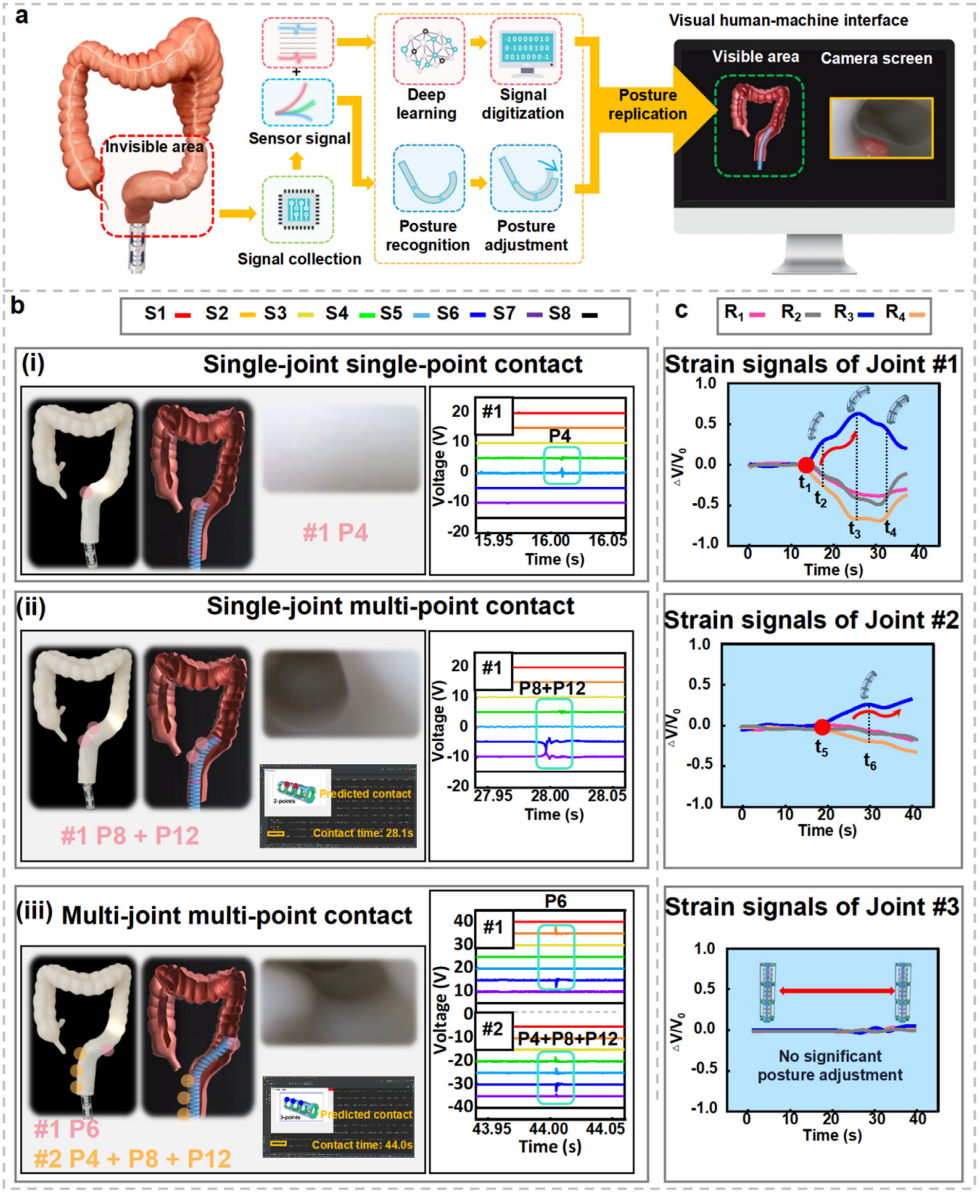
Adaptive crawling and digital‐twin‐based 3D posture visualization interface of continuum robot within silicone intestine model. a) Process of the digital‐twin‐based 3D posture visualization interface for continuum robot. b) Comparison between invisibility and visualization of continuum robot postures, along with display of tactile sensing points, under the scenarios of b‐i) single‐joint single‐point contact, b‐ii) single‐joint multi‐point contact, and b‐iii) multi‐joint multi‐point contact. c) Display of strain sensing signals of the continuum robot during adaptive crawling through silicone intestine model.

During colonoscopy, surgeons rely on colonoscope camera visuals and experience to locate lesions owing to the robot's invisible internal position, raising safety and accuracy concerns. To address this issue, a digital‐twin‐based 3D posture visualization interface for a continuum robot was developed based on the Unity 3D platform, utilizing the bimodal information from the E‐armor to reproduce the tactile and strain sensing states of the continuum robot, as well as its bending posture in the intestine. The digital tactile signals of this system could be used to identify contact points based on deep learning algorithms and form feedback control instructions. The detected strain sensing signals could identify the bending posture of the continuum robot, enabling automatic adjustment of the robot's forward posture based on compliance control strategies. Then, the data from these bimodal sensing information could then be processed using a smoothing algorithm to display virtual images of the continuum robot's posture. Therefore, the digital‐twin‐based 3D posture visualization interface of the continuum robot and the front‐end camera captured video enrich the information of human–robot visual interaction and improved the safety of colonoscopy operations (**Figure** **5**a). The crawling of the continuum robot in a silicone intestine model, which included three typical contact states: single‐joint single‐point contact, single‐joint multi‐point contact, and multi‐joint multi‐point contact, was depicted (Figure 5b). A single‐point contact event occurred for the continuum robot at P4 of Joint #1, and the signal sequence showed the tactile TENG signals of S4 and S5 for the identification of P4. According to the compliance control strategy, the robot proceeded forward with a rightward bending (Figure 5b‐i). As the robot moved forward, Joint #1 began to curve within the sigmoid colon's frontend, and the signal sequence indicated the coupled signal of S4 with simple signals of S6 and S7 (Figure 5b‐ii). Simultaneous contacts of P8 and P12 were observed during the bending process. The strain curves of *R*_3_ and *R*_4_ indicated the bending of the sigmoid rectum for Joint #1 and rightward bending for Joint #2. As Joint #2 entered the intestine, the forefront of Joint #1 reached the sigmoid rectum. At the moment when P6 of Joint #1 and P4, P8, and P12 of Joint #2 contacted simultaneously, the signal sequence showed that the E‐armor unit on Joint #2 transmitted a coupled signal through S4; while S5, S6, and S7 transmitted simple signals (Figure 5b‐iii). Due to the narrow interior of the silicone intestine and irregular sidewalls, multi‐point contact events occurred more frequently, making the tactile recognition more critical. This work underscored the potential application of a digital‐twin‐based visual interface, addressing its visibility constraints. Coupled with the front‐end camera, this platform profoundly augmented the surgical safety of colonoscopy. The strain signals from the E‐armor unit on each joint during the intervention of the robot in the silicone intestine model were illustrated (Figure 5c). These signals were fed into Unity 3D to visualize the bending angles of the robot's joints. The digital‐twin‐based 3D posture visualization interface of the continuum robot was filmed (Movie S4, Supporting Information).

## 4 Conclusion

This study introduces a bimodal stretchable E‐armor resembling artificial synapses, featuring a 3D crosslinked sensing‐net structure designed for full coverage of a colonoscopic continuum robot exceeding 300 mm in length. The E‐armor incorporates 48 tactile sensing points and 12 strain sensing edges at the joints of the continuum robot, all while requiring only 48 connecting wires. It serves the dual purpose of providing tactile sensing for the robot's surface and strain sensing for its body posture. Structurally, the design employs a separated substrate and a bilayer co‐electrode strategy, which minimize circuit layout density and prevent signal crosstalk, enhancing its suitability for integration within the robot's interior. The innovative use of TIFs with alternating positive and negative characteristics enriches the signal features and facilitates the conversion of TENG signals into digital encoding. To address the complexities and potential signal weakening due to coupling on the co‐electrode, a recognition strategy based on the CNN‐LSTM framework has been developed, achieving an impressive accuracy of 99.31%. The strain sensing elements of the E‐armor are fabricated using SA/PAM/NaCl conductive hydrogel, along with a tailored recognition strategy for identifying the continuum robot's posture. A compliance control strategy for the continuum robot leverages the synergy between tactile and strain information from the E‐armor, optimizing bending decisions. An intestinal‐shaped pipeline is constructed as an adaptive navigation environment for the robot, which successfully completes an adaptive navigation demonstration in this setting. This showcases the sensitivity, stability, and reliability of the E‐armor, as well as the effectiveness of the compliance control strategy. Finally, a 3D posture visualization interface based on digital twin technology is developed to record and reproduce the contact events and posture changes of the continuum robot during its navigation in an invisible intestinal environment. This work illustrates that the 3D structured, stretchable E‐armor fulfills the perception requirements of the continuum robot, providing bimodal sensing capabilities and demonstrating significant potential in the field of intelligent colonoscopy.

## 5 Experimental Section

### Materials

Sodium alginate (SA), acrylamide (AM), sodium chloride (NaCl), *N*, *N*’‐methylene‐bisacrylamide (MBAA), n‐hexane, benzophenone (BP), and ethanol were purchased from Shanghai Aladdin Biochemical Technology Co., Ltd. Irgacure 2959 was purchased from Shanghai Huayueye Technology Co., Ltd. Ecoflex was purchased from Shanghai Dongzhixuan Co., Ltd. Dowsil 734 was purchased from Shanghai Songjin Co., Ltd. PA particulate, PVA particulate, and PTFE particulate were purchased from Kexinda Polymer Materials Co., Ltd.

### Modification of Ecoflex's Electronegativity

i) PA particulate (diameter 200 µm), PVA particulate (diameter 90 µm), and PTFE particulate (diameter 0.1 µm) were mixed with Ecoflex prepolymer at a mass ratio of 2:10 each. The mixture was stirred for 2 min. ii) 0.5 mL of n‐hexane was added; then, the mixture was dispersed using a JY92‐IIDN ultrasonic cell disruptor at a 15% dispersion power for 2 min.

### Fabrication of Sensing Belt

i) After evacuating the mixed solutions for 5 min, each solution was drawn using a pipette and dropped into corresponding positions of the mold. ii) The solutions were evacuated again for 2 min; then heated at 80 °C for 20 min. iii) The products were demolded to obtain the TIFs. iv) Using Ecoflex as the prepolymer, steps i)–iii) were repeated to fabricate the Ecoflex flexible substrate. v) Surface hydrophilicity treatment was performed on the Ecoflex flexible substrate: the substrate was placed in an oxygen plasma cleaner (PDC‐MG, Mingheng Co., Ltd.) with the channel side facing upward and treated at 250 W for 20 min. vi) Surface hydrogen bonding treatment was performed on the Ecoflex flexible substrate and TIF: the Ecoflex flexible substrate and TIF were immersed in BP solution (10 wt% in ethanol) for 2 min; then, dried with nitrogen gas. vii) The hydrogel solution was prepared: SA particles were added to the NaCl solution. The mixture was stirred at room temperature using a magnetic stirrer (SN‐MSY‐2HD, SUNNE Co., Ltd.) for 8 h to fully swell SA in the NaCl solution. Manual stirring continued at 50 °C for 0.5 h to dissolve SA completely. A transparent and uniformly stable NaCl/SA composite solution was obtained and allowed to sit overnight at room temperature. AM monomer was added and stirred for 2 h. An ice‐water bath was performed on the solution. Crosslinker MBAA and Irgacure 2959 were added, stirred for 10 min, then centrifuged for 10 min to obtain a uniformly transparent solution. viii) The hydrogel solution was injected into the channels of the Ecoflex substrate. An isolating film was placed on the hydrogel surface, and a UV lamp (power 20 W, Gaodeng Co., Ltd.) with a wavelength of 365 nm was used to irradiate for 10 min. The hydrogel solution was injected on the surface of the isolation layer again, the surface of the hydrogel was covered with TIF, and the above ultraviolet curing operation was repeated.

### Fabrication of Sensing Ring

i) Similar to the sensing belt, PA and PTFE particulate were mixed with Ecoflex prepolymer. Procedures i)–iii) described in the previous section were followed to obtain the TIF of the sensing ring. ii) A 3D printer was used to fabricate supporting rings using soft resin material. iii) Al electrodes were attached to the inner diameter of the supporting rings. iv) After pre‐stretching the TIF, it was attached to the aluminum electrodes. TIF was fixed with Al electrode using Dowsil 734.

### Assembly of the E‐Armor

i) The four sensing rings were arranged in a specific order. ii) A small amount of adhesive was used to attach the four sensing belts in the correct sequence to the outer diameter of the supporting rings. A layer of transparent tape was used at the tactile sensing points between each sensing belt and the supporting ring to enhance the rebound ability of the sensing belts. iii) Finally, an anti‐static oil was applied to the outer surface of the sensing belt and the inner surface of the PVA‐Ecoflex part to minimize external static interference and charge crosstalk between different tactile sensing points in the internal environment.

### Layout of the E‐Armor

In this process, two signal wires were led out from both ends of the sensing belt, and one signal wire was led out from the lower sensing layer, totaling 12 wires. The signal wires were fixed using Dowsil 734. Shielded wires with a diameter of 0.05 mm were chosen as the signal wires to minimize electrical interference in subsequent circuits. Refer to Figure S24, Supporting Information for a detailed schematic of the process.

### Statistical Analysis

Quantitative results are presented as mean ± standard deviation (SD) throughout this study. Error bars in graphical representations reflect variability across duplicate experimental measurements for all tested samples, ensuring reproducibility. Data analysis workflows integrated Matlab R2023a (for statistical modeling and signal processing), Microsoft Excel 2021 (for raw data organization and preliminary calculations), and Origin 2022 (for graphical visualization and curve fitting).

## Conflict of Interest

The authors declare no conflict of interest.

## Author Contributions

Y.S., T.C., and D.L. contributed equally to this work. Y.S., T.C., H.L., and C.L. conceived the idea. Y.S., H.L., and T.J. planned and performed the experiments. Y.S. designed and prepared the E‐armor. H.L. designed and built the continuum robot system. Y.S. took all the photos shown in the Figures. D.L. wrote the control programs and algorithms for demonstration. F.W. and L.S. provided some chemical materials and experimental equipment. Y.S. and D.L. analyzed the data and drafted the manuscript. Y.S., D.L., and H.L. edited the manuscript.

## Supporting information



Supporting Information

Supplemental Movie 1

Supplemental Movie 2

Supplemental Movie 3

Supplemental Movie 4

## Data Availability

The data that support the findings of this study are available from the corresponding author upon reasonable request.
