# Supplementary material for: Stretchable, Multiplexed, and Bimodal Sensing Electronic Armor for Colonoscopic Continuum Robot Enhanced by Triboelectric Artificial Synapse
Source: Adv Mater. 2025 Jun 5;37(33):2502203. doi: 10.1002/adma.202502203 (PMC12369696; doi:10.1002/adma.202502203)
Supplement: Supplementary file 1 — Supporting Information [file ADMA-37-2502203-s001.docx]

Supporting Information

Stretchable, multiplexed, and bimodal sensing electronic armor for colonoscopic continuum robot enhanced by triboelectric artificial synapse

Yuyang Sun, Tao Chen, Dongsheng Li, Hanyang Li, Tianci Ji, Fengxia Wang, Lining Sun, Chengkuo Lee^*^, and Huicong Liu^*^

Y. Sun, D. Li, H. Li, T. Ji, F. Wang, L. Sun, H. Liu

School of Mechanical and Electrical Engineering, Jiangsu Key Laboratory of Embodied Intelligence Robot Technology, Soochow University, Suzhou, 215006, China
E-mail: [hcliu078@suda.edu.cn](mailto:hcliu078@suda.edu.cn)

C. Lee

Department of Electrical & Computer Engineering, National University of Singapore, Singapore 117576, Singapore.

E-mail: [elelc@nus.edu.sg](mailto:elelc@nus.edu.sg)

T. Chen

School of future science and engineering, Soochow University, Suzhou, 215200, China.

Y. Sun, T. Chen and D. Li contributed equally to this work.

This file includes:

**Supporting Texts**

Text S1: Biocompatibility validation of the flexible materials.

Text S2: Detailed description of the continuum robot’s design and structure.

Text S3: Construction of the control system for the continuum robot.

Text S4: Tactile sensing performance of the E-armor unit.

Text S5: Contact testing of E-armor with materials of varying stiffnesses levels.

Text S6: Tactile sensing performance of E-armor under varying pH conditions.

Text S7: Supporting explanation of the digitization encoding strategy for E-armor tactile sensing signals.

Text S8: Description of the digital encoding strategy workflow.

Text S9: Structural framework for CNN-LSTM network and confusion. matrices of recognition accuracies of different methods.

Text S10: Comparative analysis of algorithm performance.

Text S11: Formula derivation of strain sensing mechanism.

Text S12: Strain sensing performance of E-armor under varying pH conditions.

Text S13: Principle of strain signal amplifier

Text S14: Softness of the continuum robot integrated with E-armor.

Text S15: Compliance control experimental demonstration of the continuum robot.

Text S16: Compliance control strategy generalizable analysis.

Text S17: Compliance control strategy of a multi-joint continuum robot.

Text S18: Closed-Loop Shape Control Strategy for Continuum Robot.

Text S19: Compliance control strategy and autonomous navigation of a continuum robot integrated with E-armor in an intestine-shaped acrylic pipeline.

**Supporting Figures**

Figure S1: Schematic of the components of the E-armor unit.

Figure S2: Detailed description of the SA/PAM/NaCl hydrogel.

Figure S3. Cell viability rates of flexible materials.

Figure S4. Live/dead fluorescence staining of flexible materials.

Figure S5: Detailed description of the continuum robot’s design and structure.

Figure S6: Construction of the control system for the continuum robot.

Figure S7: Tactile sensing performance of the E-armor unit.

Figure S8. Triboelectric signals of E-armor in contact with nine simulated tissue samples.

Figure S9. Open-circuit voltage of triboelectric signals from E-armor in varied pH environments.

Figure S10: Supporting explanation of the digitization encoding strategy for E-armor tactile sensing signals.

Figure S11. Digital encoding strategy workflow.

Figure S12: The waveforms of simple signals, coupled signals, and theoretical signals under different contact.

Figure S23: Structural framework for CNN-LSTM network and confusion matrices of recognition accuracies of different methods.

Figure S14: Supporting performance testing of the strain sensing.

Figure S15. Strain signals of E-Armor in varied pH environments.

Figure S16: The strain signal amplifier circuit.

Figure S17: Compliance control experimental demonstration of the continuum robot.

Figure S18. Relationship between E-armor’s strain signals and the continuum robot's joint bending angles.

Figure S19. Relationship between E-armor’s strain signals and the continuum robot’s joint bending angles.

Figure S20. Geometric analysis of the forward kinematics of continuum robot

Figure S21. Schematic of the continuum robot coordinate system for the single-bending joint.

Figure S22: Compliance control strategy of multi-joint continuum robot.

Figure S23 Flowchart of the bending shape control algorithm for the continuum robot.

Figure S24: Compliance control strategy and autonomous navigation of a continuum robot integrated with E-armor in an intestine-shaped acrylic pipeline.

Figure S25: The preparation process of E-armor.

**Supporting Table**

Table S1: Key materials of CCK-8 assay

Table S2: Cell viability rates of flexible materials

Table S3: Key materials of live/dead fluorescence staining assay

Table S4: Parameters of the CNN-LSTM network structure

Table S5 The training and testing time of all compared method

Table S6: Elastic modulus of E-armor materials

Table S7: Structure parameters of the continuum robot

Table S8: D-H parameters table of the basic bending unit

Table S9: Comparison of performance of triboelectric sensor arrays based on co-electrode structure

Table S10: Comparison of the performance of continuum robots integrated with electronic skins

Table S11: Autonomous posture adjustment strategy of continuous robots

**Supporting Movies**

Movie S1: Posture recognition of single-joint, and multi-joint configurations in continuum robots using the E-armor.

Movie S2: Demonstration of compliance control of the continuum robot in pipelines with bending angles of 90°, 110° and 130°.

Movie S3: Autonomous navigation of a continuum robot integrated with E-armor in an intestine-shaped acrylic pipeline.

Movie S4: Digital-twin-based 3D posture visualization interface of continuum robot.

**Figure S1. Schematic of the components of the E-armor unit.** a) Schematic of the E-armor unit. i) Schematic of the sensing belt. ii) Schematic of the sensing ring. b) Image of the components of E-armor. c) Schematic of the E-armor integrated with the continuum robot. d) Schematic of the triboelectric interlinked film and the SEM images of PA-Ecoflex, PVA-Ecoflex and PTFE-Ecoflex.

**Figure S2. Detailed description of the SA/PAM/NaCl hydrogel.** a) The structure of SA/PAM/NaCl hydrogel. b) The molecular formula of SA/PAM/NaCl hydrogel. c) The transmittance of SA/PAM/NaCl hydrogel. d) The stretched state of SA/PAM/NaCl hydrogel. e) The SEM images of SA/PAM/NaCl hydrogel.

**Text S1: Biocompatibility validation of the flexible materials**

The CCK-8 assay was performed to quantitatively measure cellular metabolic activity and detect potential cytotoxic effects of material extracts, while the Live/Dead assay provided direct visual evidence of membrane integrity preservation through fluorescent imaging of cell distributions. These complementary assays collectively validate the biosafety of our E-armor components under physiological conditions.

**1.CCK-8 assay for material biocompatibility**

1.1 Materials and equipment

Table S1 delineates the key materials utilized in the CCK-8 assay.

**Table S1: Key materials of CCK-8 assay**

| **Experimental Materials** | **Brand/Catalog No.** |
| --- | --- |
| MEM medium | Biosharp China (BL307A) |
| Fetal bovine serum (FBS) | Opcell China (BS-1105) |
| Trypsin digestion solution | Beyotime China (C0201) |
| Penicillin-streptomycin (P/S) | Beyotime China (C0222) |
| PBS | Beyotime China (C0221A) |
| CCK-8 assay kit | Beyotime China (C0037) |
| Cell culture plate | NEST China |
| CO₂ incubator | Shanghai Yiheng China (BPN-80CH UV) |
| Inverted fluorescence microscope | Aopu China (DSZ2000X) |
| Microplate reader | Thermo USA (MK3) |

1.2.Experimental methods

1.2.1 Experimental groups

Six test groups were fabricated, comprising: (1) Ecoflex; (2) PA-Ecoflex; (3) PVA-Ecoflex; (4) PTFE-Ecoflex; (5) Hydrogel; and (6) Control group, with triplicate wells prepared for each group.

1.2.2 Experimental procedure

(1) Sample preparation

The test samples were prepared as follows: UV sterilization for 15 minutes on each side, immersion in 75% ethanol for 10 minutes, followed by three washes with PBS (5 minutes each). The samples were then extracted in complete medium at a ratio of 0.1 g/mL and incubated at 37°C for 24 hours. Complete medium extracted under identical conditions served as the normal control group medium. After extraction, the extract was aspirated, and the volume was adjusted for testing.

(2) Cell seeding

L929 cell suspension, prepared by resuspending cells (passage ≥2) adapted in complete medium (89% MEM + 10% FBS + 1% P/S), was seeded into a 96-well plate at a density of 1×105 cells/mL (0.1 mL per well). The no-sample group, inoculated with an equal number of cells, served as the control group. The no-cell and no-sample group, inoculated with an equal volume of medium, was designated as the blank group. Peripheral wells were filled with PBS, and the plate was cultured overnight.

(3) Treatment

The original medium was aspirated and replaced with the corresponding sample extracts for each group. Simultaneously, 2-fold and 4-fold diluted extract groups were prepared for each sample. The cultures were then continued for 24 hours.

(4) Detection

The original medium was removed and 100 μL of CCK8 working solution (90% MEM + 10% CCK8 detection reagent) was added to each well. Wells without cells served as blank controls. After appropriate incubation, the absorbance at 450 nm was measured using a microplate reader.

(5) Data analysis

Based on the absorbance values, the cell viability rate and inter-group differences were calculated for each group. The calculation formula is as follows:

Cell Viability (%) = 100% × (ODSample - ODBlank) / (ODControl - ODBlank)

(6) Result evaluation

The cytotoxicity of test samples was determined based on cell morphology observation and cell viability test results.

Quantitative evaluation criteria: Lower viability indicates higher potential cytotoxicity of the test sample. When the viability drops below 70% of the Control group, the sample is considered to have potential cytotoxicity.

1.3.Experimental results and analysis

The test results demonstrate that the extract-treated groups of Ecoflex, PA-Ecoflex, PVA-Ecoflex, PTFE-Ecoflex, and Hydrogel all exhibited cell viability rates exceeding 70% of the Control group, with no significant differences compared to the Control group. Under these testing conditions, none of these samples showed potential cytotoxicity. Table S2 presents the cell viability rates of each sample group relative to the Control group. As shown in Figure S3, although the cell viability rate of the hydrogel sample is relatively low, it still remains above 70%, demonstrating that the hydrogel has been confirmed to possess sufficient biocompatibility.

**Table S2: Cell viability rates of flexible materials**

| **Sample** | **Quantitative assessment results** | |
| --- | --- | --- |
|  | **Cell viability (%)** | **SD (%)** |
| Ecoflex | 97.43 | 2.74 |
| PA-Ecoflex | 99.16 | 4.25 |
| PVA-Ecoflex | 101.79 | 3.68 |
| PTFE-Ecoflex | 97.43 | 4.73 |
| Hydrogel | 72.65 | 0.88 |

**Figure S3. Cell viability rates of flexible materials.**

**2.Live/dead fluorescence staining for material biocompatibility**

2.1 Materials and equipment

Table S3 delineates the key materials utilized in the live/dead fluorescence staining.

**Table S3: Key materials of live/dead fluorescence staining assay**

| **Experimental Materials** | **Brand/Catalog No.** |
| --- | --- |
| MEM medium | Biosharp China (BL307A) |
| Fetal bovine serum (FBS) | Opcell China (BS-1105) |
| Trypsin digestion solution | Beyotime China (C0201) |
| Penicillin-streptomycin (P/S) | Beyotime China (C0222) |
| PBS | Beyotime China (C0221A) |
| Cell culture plate | NEST China |
| Calcein/PI cell viability & Cytotoxicity assay kit | Beyotime China (C2015) |
| CO₂ incubator | Shanghai Yiheng China (BPN-80CH UV) |
| Inverted biological microscope | Aopu China (DSZ2000X) |
| Microplate reader | Thermo USA (MK3) |

2.2 Experimental methods

2.2.1 Experimental groups

Six test groups were fabricated, comprising: (1) Ecoflex; (2) PA-Ecoflex; (3) PVA-Ecoflex; (4) PTFE-Ecoflex; (5) Hydrogel; and (6) Control group, with no replicate wells prepared for each group.

2.2 Experimental procedure

(1) Sample preparation

Extract preparation of test samples was performed following the same method as the CCK-8 assay.

(2) Cell seeding

L929 cells (passage≥2) were suspended in complete medium (89% MEM + 10% FBS + 1% P/S) and seeded into a 24-well plate at a density of 1×10⁵ cells/mL (0.5 mL per well), followed by 24 hour incubation.

(3) Treatment

The original medium was removed, and the sample extracts from each group were added accordingly. The cells were cultured for an additional 24 hours.

(4) Incubation

The original culture medium was aspirated, and the cells were washed once with PBS. An appropriate volume of Calcein AM/PI detection working solution was added to cover the samples, followed by incubation at 37°C in the dark for 30 minutes. The staining solution was then discarded, and the cells were washed 2-3 times with PBS.

(5) Imaging

The staining results were observed under a fluorescence microscope (Calcein AM shows green fluorescence, Ex/Em=494/517nm; PI shows red fluorescence, Ex/Em=535/617nm).

2.3Experimental results

The imaging results are shown in the Figure S4. The dense green fluorescence signals (Calcein-AM) observed across the five material groups demonstrate statistically significant biocompatibility, with signal intensity exceeding ISO 10993-5 thresholds (viability >70%) in all test cohorts.

**Figure S4. Live/dead fluorescence staining of flexible materials.**

**Text S2: Detailed description of the continuum robot’s design and structure**

The continuum robot consists of bending units made up of supporting discs, each with a diameter of 14 mm and a thickness of 7 mm. Figure S5a(i) illustrates the structure of bending unit. Nickel-titanium alloy elastic wires were used to connect adjacent supporting discs, thereby forming a bending unit with two degrees of freedom. In addition, two joint anchor holes were positioned on each end face of the supporting disc, allowing for the fixation of the nickel-titanium alloy elastic wires, while the wire holes were used to secure the drive wires, as shown in Figure S5a(ii). Figure S5a(iii) depicts an assembly of a continuum robot, consisting of four interconnected joints, each joint composed of 4 bend units.

In this study, the continuum robot was employed with a dual-driving mode, using a tendon-driven approach for motion transmission. As depicted in Figure S5b(i), the dual-driving module is illustrated. This module predominantly comprised bearings, winding spool shaft, drive wires, winding spool, coupling, and DC servo motor. The interaction between the winding spool and its shaft was achieved through a clearance fit. Two fastening screw holes were provided on the winding spool for securing it onto the winding spool shaft. One end of the winding spool shaft was fixed to a flange plate via a bearing, while the other end was connected to the DC servo motor through a coupling. Each bending degree of freedom of the continuum robot was driven by a motor that controlled the contraction or extension of a group of drive wires, wound around a winding spool. As shown in Figure S5b(ii), the tendon-driven structure is demonstrated. One end of the drive wire was wound around the winding spool, while the other end was threaded through a spring bushing with an outer diameter of 0.8 mm and an inner diameter of 0.4 mm, and then fixed onto a supporting disc.

As shown in Figure S5c, the schematic represents the structure of the driving apparatus. Eight dual-driving modules were evenly distributed and fixed onto a flange plate at equidistant angles. The three flange plates were interconnected by double-ended screws, forming the main body of the driving apparatus. Flange plates 1 and 3 were securely affixed to two supporting brackets using bearings. A stepper motor was connected to the main body of the driving apparatus through a pair of gears, allowing the apparatus to rotate about its own axis. Simultaneously, the stepper motor on the lower linear rail was responsible for the forward and backward feed of the entire device. The entire driving apparatus was placed on a baseplate, which was securely fixed onto a long-stroke linear rail. This setup granted the continuum robot body the freedom to move forward and backward. Simultaneously, to ensure the stability of the driving apparatus during its motion, two optical axis linear rails were positioned on the underside of the baseplate. The arrangement of the robot is depicted in Figure S5d(i). Figure S5d(ii) illustrates the in space, showcasing its remarkable reliability and flexibility.

**Figure S5. Detailed description of the continuum robot’s design and structure.** a) Design and structure of the continuum robot bending unit. i) Schematic of the bending unit structure. ii) Schematic of the supporting disc structure. iii) Schematic of the assembled continuum robot. b) Schematic of the driven module. i) Schematic of the dual-driving module. ii) Schematic of the tendon-driven structure. c) Schematic of the driving apparatus. d) Schematic of the structure of the continuum robot. i) Arrangement of the continuum robot. ii) The bending posture of the continuum robot in space.

**Text S3: Construction of the control system for the continuum robot**

The overall architecture of the continuum robot control system, as depicted in Figure S6a, consists primarily of three components: the master control end, the drive end, and the execution end. The master control end was a PC. During the colonoscopy examination performed by the continuum robot system, the PC served as the upper-level controller and receives information from the continuum robot through the USB serial communication protocol.

The drive end primarily consisted of a controller, drivers, DC servo motors, and stepper motors. Different types of motors required different controllers. Among these, stepper motors were responsible for the overall forward and twisting motion of the robot. The SMC606 six-axis motion control card from LSI Logic was used in combination with the DM542 driver to control the stepper motors. The SMC606 was a six-axis motion control card based on an embedded processor and FPGA hardware architecture, and it could communicate with the PC host via industrial Ethernet interface protocol. DC servo motors were of the RE13 series produced by Swiss company Maxon. These motors had an outer diameter of 13 mm and were equipped with a 131:1 planetary gear reducer and a dual-channel 256-line encoder. These motors primarily drove the bending of various segments of the continuum robot’s body. The motor controller chosen was the EPOS2 24/2 integrated positioning controller from Maxon. It could communicate with the host PC through protocols like USB and CAN. Adjacent controllers were interconnected through the CAN bus. Motion control for the motors could be achieved by calling relevant library functions from the controller’s dynamic link library.

The execution end primarily consisted of the continuum robot, the E-armor integrated on its surface, a camera integrated at the front end of the continuum robot, and a data acquisition card (ART Technology, USB5630) used for real-time sensor data collection. The data acquisition card could transmit the collected sensor feedback information to the PC at the master control end via USB serial communication. The camera provided visual feedback to the operator through the display monitor at the master control end, displaying real-time visual images from the front end of the continuum robot. The refresh rate for visual feedback, determined by the frame capture rate of the camera, was set to 5 ms in the program.

The constructed continuum robotic system platform utilized a 24 V DC power supply for both controllers and lower-level motor drivers, while a 5 V DC power supply was used for the sensor circuit. In the process of building the prototype of the continuum robotic system, eight dual-driving modules were evenly distributed in a circular pattern, as shown in Figure S6b. During the control of the robot’s bending, the continuum robot motion control software developed on the PC sent motion control commands to the SMC606 motion control card and EPOS2 24/2 controller via Ethernet and USB communication protocols. When the EPOS2 24/2 controller received the motion control commands from the PC, it communicated with the DC servo motors through its motor encoder interface. This caused the motors to rotate the winding spools via couplings. By adjusting the length of the driving wires, each joint of the robot was bent into the corresponding shape.

**Figure S6. Construction of the control system for the continuum robot.** a) The overall architecture of the designed continuum robot control system. b) Assembly of the prototype of the continuum robot control system.

**Text S4: Tactile sensing performance of the E-armor unit**

To further substantiate the existence of electrostatic induction between PTFE-Ecoflex and PA-Ecoflex, the potential distribution of a TENG pair during their approach was simulated in four stages using finite element simulation software (COMSOL) (Figure S7a). When the two are 10 mm apart, the potential difference between them reaches its maximum. As PTFE-Ecoflex approaches PA-Ecoflex, the potential difference in the electrode gradually decreases. The change in the potential difference in the electrode drives free electrons to flow between the ground and the electrodes, thereby generating an alternating current signal. During the material preparation process, it is necessary to explore the optimal mass ratio of PTFE/PA to Ecoflex in order to obtain a TENG pair with the best triboelectric performance. Three TENG pairs were prepared with PTFE/PA and Ecoflex mass ratios of 1:10, 2:10, and 3:10, respectively. With the increase of doped particles (PA and PTFE), the open-circuit voltages generated by the TENG pair during contact. However, beyond a ratio of 2:10, the increase in open-circuit voltage was no longer significant, hence a mass ratio of 2:10 was chosen, as shown in Figure S7b. To investigate whether the open-circuit voltage of the TENG pair would show significant differences when in contact with different external materials, experiments were conducted using human skin, wood, and plastic as test materials. The results indicated that under a contact pressure of 1 kPa, the open-circuit voltage generated by TENG pairs in all three situations remained consistent, as shown in Figure S7c. This confirms that the TENG pair based on the bilayer co-electrode structure can produce stable signals regardless of the contact materials. To avoid the influence of strain on tactile sensing performance, the interaction was explored under different strain of 10%, 50%, and 100%. The results showed stable open-circuit voltage between the TENG pair, as the tactile sensing points fixed on supporting rings did not stretch or affect strain, as shown in Figure S7d.

The tactile sensing points of E-armor are responsible for perceiving external tactile sensations. Placed the TENG pair under different contact pressures ranging from 0.5 to 7 kPa and collected the open-circuit voltage (Figure S7e(i)). As the pressure increases, the open-circuit voltage of PA-Ecoflex of TENG pair gradually increases, with the trough values falling from -1.41 to -4.61 V and the peak values rising from 0.89 to 3.71 V. The open-circuit voltage of PTFE-Ecoflex increases as well, with the peak values rising from 1.39 to 4.62 V and the trough values falling from -0.87 to -3.71 V. After 5 kPa, the rate of increase/decrease gradually slows down and tends to stabilize. This is because the increased pressure to the TENG pair is related to the larger deformation of the tactile sensing hemispheres, leading to a larger contact area between PA-Ecoflex and PTFE-Ecoflex sensing surfaces. The increased amount of charge transfer results in a larger voltage output. However, when the pressure increases to a certain extent, the tactile sensing hemispheres reach maximum deformation, and the contact area does not increase significantly, resulting in a saturation of the output voltage. Clearly display the open-circuit voltage waveform of the TENG pair under a pressure of 3 kPa. It can be seen that the voltage waveform of PA-Ecoflex shows opposite trends to PTFE-Ecoflex in relation to the peaks and troughs (Figure S7e(ii)-(iii)). Specifically, the voltage signal troughs of PA-Ecoflex coincide with the appearance of the voltage signal peaks of PTFE-Ecoflex, and the absolute values of the signal troughs are equal to or close to those of the signal peaks. Similarly, the signal peaks of PA-Ecoflex and the signal troughs of PTFE-Ecoflex appear at the same time, and the absolute voltage values are close to or equal to each other. The voltage signal of PA-Ecoflex presents a characteristic of first appearing troughs then peaks, while the voltage signal of PTFE-Ecoflex shows an opposite characteristic of first peaks then troughs. This indicates that during the contact process, the charges are transferred between the two surfaces without significant charge loss, which confirms that the structural design concept of the TENG pair is rational.

The open-circuit voltage transmitted by the TENG pair under a pressure of 0.5 kPa was obtained by contacting it at different frequencies (Figure S7f(i)). As the frequency increases from 0.5 to 5 Hz, the voltage trough values of PA-Ecoflex of the TENG pair decrease from -1.26 to -9.12 V, and the voltage peak values rise from 0.75 to 7.15 V. Conversely, the voltage peak values of the of PTFE-Ecoflex rise from 1.03 to 9.19 V, and the trough values change from -0.93 to -7.22 V. As the PA-Ecoflex and PTFE-Ecoflex come into contact, the triboelectric charge between them increases initially and then tends to stabilize with the increase in contact frequency. This could be that as the frequency increases, the charges accumulated on the surfaces of PA-Ecoflex and PTFE-Ecoflex cannot fully neutralize, leading to a stabilizing growth in output voltage. The open-circuit voltage of the TENG pair under contact frequency of 1Hz is demonstrated clearly (Figure S7f(ii)-(iii)). The signal waveforms of PA-Ecoflex and PTFE-Ecoflex also show an opposite trend. During each contact process, the signal trough of PA-Ecoflex occurs at the same time as the signal peak of PTFE-Ecoflex, while the signal peak of PA-Ecoflex and the signal trough of PTFE-Ecoflex occur at the same time. The absolute values of each pair of signal peak and trough are the same or approximate. This indicates that even when the contact frequency is changed, it can still output stable and accurate signal pairs.

The tactile sensing performance of the TENG pair has been tested and characterized. In a TENG pair, as the PA-Ecoflex and PTFE-Ecoflex electrodes simultaneously transmit voltage signals during each contact process, the peak voltage signal of PA-Ecoflex under different pressure contacts is taken as the calibration data in this work. By linearly fitting of the voltage peaks, it can be found that the sensitivity of TENG pair is 0.59 (V/kPa) under the contact pressure of 0 to 6 kPa (Figure S7g). When the contact pressure exceeds 6 kPa, the sensitivity is reduced to 0.02 (V/kPa). To verify the repeatability, a pressure of 1 kPa was applied to the TENG pair continuously for 1000 times at a frequency of 1 Hz. It can be seen that the open-circuit voltage by the PA-Ecoflex and PTFE-Ecoflex electrodes maintained excellent stability throughout the entire process, as shown in Figure S7h.

**Figure S7. Tactile sensing performance of the E-armor unit.** a) COMSOL simulation of the potential between the PTFE-Ecoflex and PA-Ecoflex. b) The open-circuit voltage of the TENG pair prepared with doping ratios of 1:10, 2:10, and 3:10. c) The open-circuit voltage of different materials contact on the TENG pair surface. d) Open-circuit voltage at 10%, 50%, and 100% strain of the TENG pair. e) The open-circuit voltage characteristics of TENG pair under different contact pressures. i) The open-circuit voltage of TENG pair under contact pressures ranging from 0.5 to 7 kPa. ii) The open-circuit voltage waveform of PA-Ecoflex and PTFE-Ecoflex under a contact pressure of 3 kPa. iii) An enlarged view of the open-circuit voltage waveforms of PA-Ecoflex and PTFE-Ecoflex under a contact pressure of 3 kPa. f) The open-circuit voltage characteristics of TENG pair under different contact frequencies. i) The open-circuit voltage of TENG pair under contact frequencies ranging from 0.5 to 5 Hz. ii) The open-circuit voltage waveform of PA-Ecoflex and PTFE-Ecoflex under a contact frequency of 1 Hz. iii) An enlarged view of the open-circuit voltage waveforms of PA-Ecoflex and PTFE-Ecoflex under a contact frequency of 1 Hz. g) Sensitivity of the TENG pair. h) Repeatability of the TENG pair.

**Text S5: Contact testing of E-armor with materials of varying stiffness levels**

The elastic modulus of intestinal polyps ranges from 5 to 50 kPa, while early-stage intestinal tumors range from 20 to 100 kPa. In contrast, malignant intestinal tumors can exhibit an elastic modulus exceeding 100 kPa.^[1–3]^ Therefore, medical silicone was used to prepare nine simulated tissues mimicking intestinal polyps or tumors, with elastic modulus of 5, 10, 20, 30, 40, 60, 80, 100, and 200 kPa. During the test, the E-armor was secured to the testing platform, and a tensile-compression testing machine was utilized to apply repeated 0.5 kPa pressure to its surface at a frequency of 1 Hz, simulating tissue interaction. Triboelectric signals from PTFE-Ecoflex and PA-Ecoflex interfaces at designated test points were recorded using an oscilloscope.

The experimental results demonstrated that the E-armor generated stable triboelectric signals (V_OC_) when interacting with simulated tissues of varying elastic modulus (5-200 kPa). As shown in the Figure S8, both PTFE-Ecoflex and PA-Ecoflex exhibited consistent voltage outputs across all elastic modulus levels, indicating that the signal stability was unaffected by the different tissue stiffnesses. The signal stability stems from E-armor’s unique working principle: when external simulated tissues contact against E-armor’s surface, this pressure causes internal contact between the embedded PTFE-Ecoflex and PA-Ecoflex layers. Crucially, the degree of contact between these two functional layers is solely determined by the applied pressure (0.5 kPa in this case), remaining completely independent of the stiffnesses, elasticity, or material properties of the external contacting tissue. Since the applied pressure remained constant, the contact area and contatct force between these two internal materials stayed uniform, ensuring stable signal output regardless of the simulated tissue’s elastic modulus.

**Figure S8. Triboelectric signals of E-armor in contact with nine simulated tissue samples.**

**Text S6: Tactile sensing performance of E-armor under varying pH conditions**

To systematically evaluate E-armor’s tactile sensing performance across physiological pH gradients, standardized buffer solutions (pH 6.5, 7.0, 7.5) replicating colorectal pH conditions were prepared.^[4]^ Three E-armor units were surface-coated with respective solutions. Using a tension-compression tester, 0.5 N contact force was cyclically applied to designated sensing points every 12 seconds over 40 minutes. Triboelectric signals from both PA-Ecoflex and PTFE-Ecoflex interfaces were simultaneously acquired via oscilloscope, generating three characteristic signal pairs (Figure S9).

The open-circuit voltage (V_OC)_ of triboelectric signals at tactile sensing points exhibits consistent stability during the 40-minute test across pH 6.5, 7.0, and 7.5 environments. Experimental results demonstrate synchronized triggering of signal pairs from E-armor’s sensing points in all pH conditions, with maintained waveform alignment and no pattern confusion or signal crossover observed. These findings confirm E-armor’s reliable long-term signal stability under pH variations.

**Figure S9. Open-circuit voltage of triboelectric signals from E-armor in varied pH environments.**

**Text S7: Supporting explanation of the digitization encoding strategy for E-armor tactile sensing signals**

Similarly, the contact events at P6 and P15 in sequence are illustrated (Figure S10a and Figure S10b). Since P6 is located at the orthogonal intersection of outer TIF S2 and inner TIF S6, the signal of channels S2 and S6 are encoded as 1 and -1, respectively. The tactile sequence code of P6 is obtained as (0, 1, 0, 0, 0, -1, 0, 0). For the contact event at P15, only the channels of S3 and S8 output signals, which are coded as -1 and 1, respectively. Therefore, the tactile sequence of P15 is coded as (0, 0, -1, 0, 0, 0, 0, 1).

These four tactile sensing points of P3, P7, P11 and P15 share the outer TIF channel S3, hence generating a coupled signal coded as ‘n’. The separated inner TIF channels are occupied as S5, S6, S7, and S8, which can be simply coded as -1, 1, -1, 1, respectively. By combining these codes, the tactile sequence code for this four-point contact scenario is (0,0, n, 0, -1, 1, -1, 1) (Figure S10c). The signal waveforms generated at each contact are also shown in an enlarged view.

**Figure S10. Supporting explanation of the digitization encoding strategy for E-armor tactile sensing signals.** a) Digitization of tactile signals exemplified by P6. b) Digitization of tactile signals exemplified by P15. c) Digitization of tactile signals for four tactile sensing points contacts on the co-electrode.
**Text S8: Description of the digital encoding strategy workflow**

The tactile sensing process initiates when physical contact occurs between the E-armor’s surface and external objects, triggering the sensing mechanism. Upon contact, triboelectric activation begins as the opposing PTFE-Ecoflex and PA-Ecoflex layers induce directional electron transfer through interfacial friction. The generated signals propagate simultaneously via two pathways: the outer triboelectric interface (TIF) through channels S1-S4 and the inner TIF through channels S5-S8. These signals converge at co-electrodes, where spatial superposition is resolved via charge conservation principles. Next, waveform polarity (positive/negative peaks) and temporal synchronization are analyzed to extract signal features, which are digitally encoded as “1” (PTFE peak-valley waveform), “-1” (PA valley-peak waveform), or “0” (null) for single-point contacts, while multi-point interactions undergo coupled signal decomposition using co-electrode cross-correlation. The integrated signals are then processed by a CNN-LSTM deep learning framework: spatial patterns are extracted from the 8-channel signal matrix via convolutional neural networks (CNN), temporal dependencies across sequential events are modeled using long short-term memory (LSTM), and a self-attention layer prioritizes critical features for noise suppression. Finally, the system outputs decoded contact coordinates (P1-P16) mapped to the continuum robot’s surface, along with a digital tactile sequence code representing the interaction dynamics.

**Figure S11. Digital encoding strategy workflow.**

**Figure S12. The waveforms of simple signals, coupled signals, and theoretical signals under different contact.** a) The contact of two tactile sensing codes as “1” being triggered simultaneously. b) The contact of two tactile sensing codes as “-1” being triggered with a slight time difference. c) The contact of a tactile sensing code as “-1” and a tactile sensing code as “1” being triggered simultaneously. d) The contact of two tactile sensing codes as “1” being triggered with a slight time difference.

**Text S9: Structural framework for CNN-LSTM network and confusion matrices of recognition accuracies of different methods**

The confusion matrices of recognition accuracies of different methods are depicted in Figure S13b. Considering that the features of TENG signals can be confused or weakened during the coupling process, the recognition accuracy of the proposed method was excellent, demonstrating the effectiveness in pattern recognition of tactile sensing signal. An ablation experiment was conducted to evaluate the performances of different components of the proposed method.

By systematically removing certain components and observing the impact on performance, we could identify the contributions of different components to the overall success. Specifically, in comparison to individual CNN and LSTM models incorporating a self-attention mechanism, the average accuracy increased by 2.29% and 2.17%, respectively. This highlights the notable enhancement in representation capability brought by the fusion of CNN and LSTM. The three deep learning methods, compared to the traditional machine learning SVM, show accuracy improvements of 17.02%, 14.73%, and 14.55%, respectively. This can be explained by the stronger learning and expressive abilities of deep learning networks, surpassing traditional machine learning approaches under sufficient data and well-tuned hyperparameters.

**Figure S13. Structural framework for CNN-LSTM network and confusion matrices of recognition accuracies of different methods.** a) Structural framework for CNN-LSTM network. b) The confusion matrices of recognition accuracies of individual CNN, LSTM, and SVM model.

**Table S4: Parameters of the CNN-LSTM network structure.**

| **Network layer** | **Parameter** | **Activation function** | **Output size** |
| --- | --- | --- | --- |
| Input | / | / | 101*1 |
| Convolutional layer | Stride = 1; Kernel size = 32; Number of channels = 64 | ReLU | 101*64 |
| Pooling Layer | Max Pooling Size = 2 | / | 101*32 |
| Convolutional layer | Stride = 1; Kernel size = 3; Number of channels = 64 | ReLU | 32*32 |
| Convolutional layer | Stride = 1; Kernel size = 3; Number of channels = 64 | ReLU | 32*32 |
| Convolutional layer | Stride = 1; Kernel size = 3; Number of channels = 64 | ReLU | 32*32 |
| Flatten layer | / | / | 1024*1 |
| LSTM layer | Cell size = 256 | / | 256*1 |
| LSTM layer | Cell size = 128 | / | 128*1 |
| LSTM layer | Cell size = 64 | / | 64*1 |
| Self-attention layer | Initial Matrix Size = 64*64 | / | 64*1 |
| F1 | Weight = 128*128; Bias = 128 | ReLU | 128*1 |
| F2 | Weight = 128*64; Bias = 64 | ReLU | 64*1 |
| F3 | Weight = 64*4; Bias = 6 | Softmax | 4*1 |

**Text S10: Comparative analysis of algorithm performance**

**Real-Time Processing:** The testing time for all models, including the proposed CNN-LSTM, is less than 0.1 seconds. This ensures that real-time classification of test data is entirely feasible. The CNN model has the shortest testing time of 0.04s, while CNN-LSTM method is only 0.05s longer than the CNN model. This slight difference has a negligible impact on the real-time classification efficiency of the collected data, especially when the sampling frequency is as low as 0.3 Hz. While the CNN-LSTM method does not achieve the shortest testing time among the four methods compared, it demonstrates the highest accuracy rate of 99.31%, surpassing SVM, LSTM, and CNN by 17.02%, 2.17%, and 2.29%, respectively.

**Table S5 The training and testing time of all compared method**

| Method | Testing Time | Accuracy |
| --- | --- | --- |
| SVM | 0.09 s | 82.29% |
| LSTM | 0.05 s | 97.14% |
| CNN | 0.04 s | 97.02% |
| CNN-LSTM | 0.09 s | 99.31% |

**Text S11: Formula derivation of strain sensing mechanism**

Strain sensing mechanism utilizes the electrical principles of resistive sensors, thus adhering to Ohm’s Law. Calculate the relationship between the relative resistance change rate and the tensile strain of the sensing belt. When the sensing belt undergoes stretching, the area of the cross-section *A-A* decreases, changing to *A’-A’*. This process can be expressed by the following formula:

Initial resistance:

$R_{0}=\frac{\rho\cdot L_{0}}{A_{0}}$ (1)

The resistance of the sensing belt when stretched to a length of *L_1_* is:

$R_{1}=\frac{\rho\cdot L_{1}}{A_{1}}$ (2)

Where *ρ* is the resistivity of the S-hydrogel electrode, *L_0_* and *L_1_* are the lengths of the sensing belt before and after stretching, and *A_0_* and *A_1_* are the cross-sectional areas of the strain strip before and after stretching.

The volume *V* of the hydrogel electrode can be considered unchanged before and after stretching, therefore：

$V=L_{0}\cdot A_{0}=L_{1}\cdot A_{1}$ (3)

Substitute the formula (3) into formula (2):

$R_{1}=\frac{\rho\cdot L_{1}^{2}}{A_{0}L_{0}}$ (4)

According to the definition of stretching, we obtain:

$\varepsilon=\frac{L_{1}-L_{0}}{L_{0}}$ (5)

By combining formulas (4) and (5), the relationship between the rate of resistance change and the strain can be obtained, i.e.:

$\frac{\Delta R}{R_{0}}=\frac{R_{1}}{R_{0}}-1=\left(\frac{L_{1}}{L_{0}}\right)^{2}-1=\varepsilon^{2}+2\varepsilon$ (6)

From the above analysis, it can be concluded that when the sensing belt is stretched, the relative resistance change rate increases; when the sensing belt is compressed, the relative resistance change rate.

**Figure S14. Supporting performance testing of the strain sensing.** a) Relative resistance change rate to pressure interference of the sensing belt under strain. b) The tensile performance characteristics of Ecoflex, PTFE-Ecoflex, PVA-Ecoflex, and PA-Ecoflex. c) The repeatability of the sensing belt.

**Text S12: Strain sensing performance of E-armor under varying pH conditions**

To assess E-armor’s strain sensing performance under physiological pH conditions, the experimental protocol was replicated using three standardized buffer solutions (pH 6.5, 7.0, 7.5) simulating colorectal pH gradients. Each solution was uniformly coated on three E-armor units. Using a tension-compression tester, the sensing belt was cyclically stretched to 350% strain every 30 seconds over 40 minutes. Resistance changes were monitored via an LCR meter, with relative resistance change rates (*ΔR/R_0_*) during initial and final stretching phases plotted under each pH condition (Figures S15a and S15b respectively).

The E-armor’s sensing belt demonstrated consistent relative resistance change rates (*ΔR/R_0_*) during initial 350% strain stretching across pH 6.5-7.5 environments, establishing baseline comparability (Figure S15a). Post-40-minute cyclic stretching revealed pH-dependent divergence: ΔR/R₀ remained stable at pH 7.0, exhibited upward drift at pH 6.5, and downward drift at pH 7.5 (Figure S15b). Mechanistic analysis indicates NaCl electrolytes maintain stable ionization within this pH range, preserving fundamental resistance-strain linearity. The permeable Ecoflex substrate allows gradual solution penetration into its matrix and hydrogel electrodes. Under pH 6.5, carboxyl protonation in sodium alginate hydrogel slightly elevates resistance, while pH 7.5-induced deprotonation reduces resistance with potential hysteresis. Crucially, the ΔR/R₀ polarity, which serves as the control basis for continuum robots’ autonomous posture adjustment, remains unaffected by acidic/alkaline-induced signal drift. This confirms the strain sensing capability’s operational stability despite intestinal pH complexity.

**Figure S15. Strain signals of E-Armor in varied pH environments.**

**Text S13: Principle of strain signal amplifier**

The principle of strain signal amplification is as follows, essentially functioning as a low-pass filter circuit. The circuit consists of three parts, as shown in Figure S16. The first part, shown in blue, is responsible for applying a constant input voltage to the hydrogel electrode inside the E-armor. The second part, shown in orange, is an operational amplifier that ensures the current in the amplifier resistors R_1_ and R_2_ is equal to the current in the hydrogel electrode resistor Rhydrogel. The third part, shown in green, includes two resistors, R_1_ and R_2_, and a capacitor. R_1_ and the capacitor form a filter to remove interference signals such as electromagnetic waves. R_2_ is an adjustable resistor used for fine-tuning the current to accommodate hydrogel electrodes with different internal resistances. The output voltage, which represents the test result, is the sum of the voltages across R_1_ and R_2_. This circuit converts the resistive signal of the E-armor into a voltage signal.

**Figure S16. The strain signal amplifier circuit.**

**Text S14: Softness of the continuum robot integrated with E-armor**

1. **Softness of the E-armor**

Due to the fact that the E-armor is wrapped around the surface of the continuum robot, it is the part that directly contacts the intestine. When selecting materials for E-armor, the stiffness of the materials is the most important concern. In this work, the E-armor was made of soft materials such as silicone or resin. The elastic modulus of these materials was measured using a tension-compression testing machine, with results summarized in Table S6.

**Table S6: Elastic modulus of E-armor materials**

| **Material** | **Elastic modulus** |
| --- | --- |
| Ecoflex | 30 ± 2.1 kPa |
| PA-Ecoflex | 35 ± 1.6 kPa |
| PTFE-Ecoflex | 28 ± 2.2 kPa |
| SA/PAM/NaCl conductive hydrogel | 20 ± 1.8 kPa |
| Soft resin | 61 ± 1.9 kPa |

Their biomechanical properties of these materials not only pose no risk but actively protect vulnerable mucosal tissues. The specialized hydrogel-silicone composite structure further creates a “mechanical buffering interface” at the tissue surface, effectively dispersing contact stresses.

2. **Structural softness of the continuum robot**

To better clarify the structural softness of the continuum robot, the following presents the calculation methodology and detailed procedure for determining its bending stiffness. The key structural parameters of the continuum robot are first defined as follows:

**Table S7: Structure parameters of the continuum robot**

| **Parameter** | **Value** |
| --- | --- |
| Outside diameter of supporting disk | 14 mm |
| Inside diameter of supporting disk | 10 mm |
| Joint length (*L*) | 100 mm |
| Thickness of supporting disk (*t*) | 7 mm |
| Number of supporting disks (*n*) | 13 |
| Material of supporting disk | Aluminum alloy |
| Young’s modulus of supporting disk (*E*) | 69 GPa |
| Diameter of NiTi alloy elastic wire (*d*) | 1.2 mm |
| Young’s modulus of NiTi alloy elastic wire (*E*) | 35 GPa |

Bending Stiffness Theoretical Model

(1) Equivalent series spring model

The overall bending stiffness is jointly contributed by the aluminum supporting disks and NiTi alloy elastic wires, which can be approximated as springs in series:

$\frac{1}{k_{total}}=\frac{1}{k_{Al}}+\frac{1}{k_{NiTi}}$ (7)

where *k_Al_* and *k_NiTi_* need to be calculated separately.

(2) Stiffness of Aluminum supporting disks (*k_Al_*)

$k_{Al}=\frac{k_{Al,single}}{n}=\frac{E_{Al}\cdot I_{Al}}{t\cdot n}=\frac{E_{Al}\cdot\frac{\pi}{64}(d_{o}^{4}-d_{i}^{4})}{t\cdot n}=0.857(Nm/rad)$(8)

Among them,

*k_Al,single_*: Bending stiffness of a single supporting disk

*I_Al_*: Moment of inertia of supporting disk

*E_Al_*: Young’s modulus of supporting disk

*d_o_*: Outside diameter of supporting disk

*d_i_*: Inside diameter of supporting disk

*t*: Thickness of supporting disk

*n*: Number of supporting disks

(3) Stiffness of NiTi alloy elastic wires (*k_NiTi_*)

The NiTi alloy elastic wires primarily contribute torsional stiffness during bending, which can be approximated as the sum of multiple parallel wires. Total stiffness of 4 parallel NiTi alloy elastic wires:

$k_{NiTi}=4\cdot k_{NiTi,single}=4\cdot\frac{3\cdot E_{NiTi}\cdot I_{NiTi}}{L}=\frac{4\cdot3\cdot E_{NiTi}\cdot\frac{\pi}{64}d^{4}}{L}=0.428(Nm/rad)$ (9)

Among them,

*k_NiTi,single_*: Bending stiffness of a NiTi alloy elastic wire

*I_NiTi_*: Moment of inertia of a NiTi alloy elastic wire

*E_NiTi_*: Young’s modulus of a NiTi alloy elastic wire

*d*: Diameter of NiTi alloy elastic wire

*L*: Joint length

(4) Overall bending stiffness (*k_total_*)

$\frac{1}{k_{total}}=\frac{1}{k_{Al}}+\frac{1}{k_{NiTi}}=\frac{1}{0.857}+\frac{1}{0.428}=1.167+2.336=3.503$ (10)

$k_{total}=\frac{1}{3.503}=0.285(Nm/rad)$ (11)

Each joint demonstrates a bending stiffness of approximately 0.285 Nm/rad, allowing gentle conformation to intestinal walls without excessive force application.

**Text S15: Compliance control experimental demonstration of the continuum robot**

To validate the compliance control strategy, Joint #1 of the continuum robot covered with the E-armor unit had been programmed to realize autonomous navigation through pipelines with three different bending angles of 90°, 110°, and 130°. As illustrated in Figure S17, all the voltage signals from the E-armor unit channels were plotted in different colors, where the tactile sensing signals of S1 to S8 were used to detect and identify the contact points from P1 to P16, and the strain sensing signals of *R_1_* to *R_4_* were utilized for bending angle detection and posture adjustment in sequence. As shown in Figure S17a(i) to (iv), when the robot joint passed through the pipeline of 90°, the sequential contact points were P4-P6-P6-P6-P6-P4-P4-P4-P4, which were inferred by the CNN-LSTM model for tactile sensing recognition. During this process, the open-circuit voltage waveforms of the TENG pairs at P4 and P6 were detailed and enlarged, which indicated that only P4 and P6 on the E-armor unit were alternately contacted. The relative voltage change rates corresponding to the sensing belts of *R_3_* and *R_4_* showed a stepwise ascending and declining trend, respectively, indicating gradual compression and stretching of the sensing belts to accommodate the bending angle and posture of Joint #1. Thereby, the strain occurrences and posture adjustments marked with red stars were observed 9 times at each contact moment. Referring to Figure S17b(i) to (iv) and Figure S17c(i) to (iv), they respectively depicted the process of the robot joint navigating through the pipelines of 110° and 130°, with their respective sequential contact points being P4-P4-P4-P4-P4-P6-P4-P4 and P6-P4-P4. When the robot joint navigated through the two different angle pipelines, the number of contacts between the robot joint and the pipe walls were 8 and 3, respectively. As observed, the relative voltage change rates corresponding to the sensing belts of *R_3_* and *R_4_* exhibited stepwise increases and decreases 8 times and 3 times, respectively, in these two cases. As could be seen, as the angle of the pipeline increased, the number of joint adjustments decreased, indicating that simpler pipelines did not require the robot to adjust its posture more frequently.

**Figure S17. Compliance control experimental demonstration of the continuum robot.** a) Demonstration of compliance control experiments of the single-joint of continuum robot in pipelines with bending angles of 90°. i) Autonomous navigation of the continuum robot in a pipeline with a bending angle of 90°. ii) Schematic of P4 and P6 waveform in the experiment. iii) Tactile signals of the single-joint of continuum robot in a pipeline with a bending angle of 90°. iv) Strain signals of the single-joint of continuum robot in a pipeline with a bending angle of 90°. b) Demonstration of compliance control experiments of the single-joint of continuum robot in pipelines with bending angles of 110°. i) Autonomous navigation of the continuum robot in a pipeline with a bending angle of 110°. ii) Schematic of P4 and P6 waveform in the experiment. iii) Tactile signals of the single-joint of continuum robot in a pipeline with a bending angle of 110°. iv) Strain signals of the single-joint of continuum robot in a pipeline with a bending angle of 110°. c) Demonstration of compliance control experiments of the single-joint of continuum robot in pipelines with bending angles of 130°. i) Autonomous navigation of the continuum robot in a pipeline with a bending angle of 130°. ii) Schematic of P1 and P7 waveform in the experiment. iii) Tactile signals of the single-joint of continuum robot in a pipeline with a bending angle of 130°. iv) Strain signals of the single-joint of continuum robot in a pipeline with a bending angle of 130°.

**Text S16: Compliance control strategy generalizable analysis**

1. **Compliance framework of continuum robots**

The compliance control strategy achieves **sensing-driven posture adjustment** through forward and inverse kinematics, as shown in the Figure S18. **Specifically, the sensing information provided by E-armor serves as guidance for continuum robot bending.** The forward kinematics, based on Denavit-Hartenberg (D-H) parameters, calculates the end-effector position from joint angles, ensuring the robot precisely tracks intestinal curvature. The inverse kinematics maps sensing information (*ΔV/V₀*) to joint angles via driving wire length variation enabling dynamic compliance.

**Figure S18. Relationship between E-armor’s strain signals and the continuum robot’s joint bending angles.**

2. **Mapping between E-armor’s strain signals and joint bending angles**

In our work, E-armor’s strain signals are utilized to reflect the bending angles *β_segment_* of the continuum robot’s joints. We commanded the continuum robot joints to bend incrementally at 10° intervals and recorded the corresponding E-armor strain signals during each bending step, with collected data shown in Figure S19. As observed, the strain signals exhibited a linear decreasing trend during bending. A linear fitting yielded the equation y = -0.009x - 0.02, with a linearity R^2^ of 0.99. This established linear relationship thus serves as a reliable guide for monitoring and controlling the joint bending of the continuum robot.

**Figure S19. Relationship between E-armor’s strain signals and the continuum robot’s joint bending angles.**

3. **Kinematic modeling of the continuum robot**

1. Forward kinematics of continuum robot

**The forward kinematics modeling of continuum robots is to solve the mapping from their actuator space to workspace.** To achieve this mapping systematically, the Denavit-Hartenberg (D-H) parameter method provides a standardized framework for modeling the kinematic chain of continuum robots. D-H parameter method describes the motion of a basic bending unit using four kinematic parameters: link length, link twist angle, link offset, and joint angle. Here, link length refers to the length of the common perpendicular line between the axes of two adjacent supporting disks. Link twist angle represents the angle between the axes of two supporting disks. Link offset denotes the distance along the common axis direction between two adjacent supporting disks, while joint angle corresponds to the bending angle of the double-pivot joint. Based on these parameters, the D-H parameter table for a basic bending unit can be established, as shown in Table S8. All geometric analyses associated with forward kinematics are illustrated in Figure S20.

**Table S8: D-H parameters table of the basic bending unit**

| *i* | $\alpha_{i-1}$ | $a_{i-1}$ | $d_{i}$ | $\theta_{i}$ |
| --- | --- | --- | --- | --- |
| 1 | 0 | $\frac{l_{j}}{\beta_{1}}tan\frac{\beta_{1}}{2}$ | 0 | -$\beta_{1}$ |
| 2 | $-\frac{\pi}{2}$ | $\frac{l_{j}}{\beta_{1}}tan\frac{\beta_{1}}{2}+l_{d}+\frac{l_{j}}{\beta_{2}}tan\frac{\beta_{2}}{2}$ | 0 | -$\beta_{2}$ |
| 3 | $\frac{\pi}{2}$ | $l_{d}+\frac{l_{j}}{\beta_{2}}tan\frac{\beta_{2}}{2}$ | 0 | 0 |

Based on the D-H parameters, the homogeneous transformation matrices between adjacent coordinate systems within a basic bending unit can be derived:

${}_{1}^{0}{T=\left[ \begin{matrix} c\beta_{1} & \begin{matrix} s\beta_{1} & 0 \end{matrix} & \frac{l_{j}}{\beta_{1}}tan\frac{\beta_{1}}{2} \\ \begin{matrix} -s\beta_{1} \\ 0 \end{matrix} & \begin{matrix} c\beta_{1} & 0 \\ 0 & 1 \end{matrix} & \begin{matrix} 0 \\ 0 \end{matrix} \\ 0 & \begin{matrix} 0 & 0 \end{matrix} & 1 \end{matrix} \right]}$	(12)

${}_{2}^{1}{T=\left[ \begin{matrix} c\beta_{2} & \begin{matrix} s\beta_{2} & 0 \end{matrix} & \frac{l_{j}}{\beta_{1}}tan\frac{\beta_{1}}{2}+l_{d}+\frac{l_{j}}{\beta_{2}}tan\frac{\beta_{2}}{2} \\ \begin{matrix} 0 \\ s\beta_{2} \end{matrix} & \begin{matrix} 0 & 1 \\ -c\beta_{2} & 0 \end{matrix} & \begin{matrix} 0 \\ 0 \end{matrix} \\ 0 & \begin{matrix} 0 & 0 \end{matrix} & 1 \end{matrix} \right]}$	(13)

${}_{3}^{2}{T=\left[ \begin{matrix} 1 & \begin{matrix} 0 & 0 \end{matrix} & l_{d}+\frac{l_{j}}{\beta_{2}}tan\frac{\beta_{2}}{2} \\ \begin{matrix} 0 \\ 0 \end{matrix} & \begin{matrix} 0 & -1 \\ 1 & 0 \end{matrix} & \begin{matrix} 0 \\ 0 \end{matrix} \\ 0 & \begin{matrix} 0 & 0 \end{matrix} & 1 \end{matrix} \right]}$	(14)

Based on the above homogeneous transformation matrices, the homogeneous transformation matrix of a basic bending unit can be obtained as:

${}_{3}^{0}{T={}_{1}^{0}T{}_{2}^{1}T}{}_{3}^{2}T$	(15)

Based on the above equation, we have:

${}_{3}^{0}{T=\left[ \begin{matrix} c\beta_{1}c\beta_{2} & \begin{matrix} s\beta_{2} & c\beta_{1}s\beta_{2} \end{matrix} & c\beta_{1}c\beta_{2}\left( l_{d}+\frac{l_{j}}{\beta_{2}}tan\frac{\beta_{2}}{2} \right)+c\beta_{1}\left( \frac{l_{j}}{\beta_{1}}tan\frac{\beta_{1}}{2}+l_{d}+\frac{l_{j}}{\beta_{2}}tan\frac{\beta_{2}}{2} \right)+\frac{l_{j}}{\beta_{1}}tan\frac{\beta_{1}}{2} \\ \begin{matrix} -s\beta_{1}c\beta_{2} \\ s\beta_{2} \end{matrix} & \begin{matrix} c\beta_{1} & s\beta_{1}s\beta_{2} \\ -c\beta_{2} & 0 \end{matrix} & \begin{matrix} -s\beta_{1}c\beta_{2}\left( l_{d}+\frac{l_{j}}{\beta_{2}}tan\frac{\beta_{2}}{2} \right)-s\beta_{1}\left( \frac{l_{j}}{\beta_{1}}tan\frac{\beta_{1}}{2}+l_{d}+\frac{l_{j}}{\beta_{2}}tan\frac{\beta_{2}}{2} \right) \\ s\beta_{2}\left( l_{d}+\frac{l_{j}}{\beta_{2}}tan\frac{\beta_{2}}{2} \right) \end{matrix} \\ 0 & \begin{matrix} 0 & 0 \end{matrix} & 1 \end{matrix} \right]}$	(16)

By serially connecting multiple basic bending units into a complete bending joint, the pose expression of the single-joint continuum can be derived as:

$T_{s}={({}_{3}^{0}{T)}}^{n}={({}_{1}^{0}T{}_{2}^{1}T{}_{3}^{2}T)}^{n}$	(17)

In the above equation, *n* represents the number of basic bending units contained in a single-joint continuum robot.

Similarly, by extending the single-joint to the entire continuum robot, as shown in Figure S20, the pose expression of the endpoint for a continuum robot containing four bending joints can be derived as:

$T_{continuum}=T_{s1}T_{s2}T_{s3}T_{s4}$	(18)

**Figure S20. Geometric analysis of the forward kinematics of continuum robot**

2. Inverse kinematics of continuum robot

**Inverse kinematics refers to the process of determining the bending and twist angles of each joint in a continuum robot based on the known end-effector position, thereby calculating the changes in the driving wire lengths within each joint. This further enables the determination of the required angular displacement of the motors.** To solve the inverse kinematics for a single bending joint of a continuum robot, one should first analyze the mapping relationship between the twist angle *θ_segment_* and bending angle *β_segment_* of a fundamental bending unit and the two joint angles *β_1_*, *β_2_*, as shown in Figure S21. Only then can the inverse kinematics be performed.

**Figure S21. Schematic of the continuum robot coordinate system for the single-bending joint.**

As shown in Figure R21, the twist angle *θ_segment_* of each fundamental bending unit aligns with the twist angle *θ_section_* of the entire bending joint. Meanwhile, the bending angle *β_segment_* of each fundamental bending unit equally divides the section’s total bending angle *β_section_*, as expressed in Equations (19) and (20):

$\theta_{segment}=\theta_{section}=arctan\frac{Z_{p}}{Y_{p}}$	(19)

$\beta_{segment}=\frac{\beta_{section}}{n}=\frac{2}{n}arctan\frac{\sqrt{Y_{p}^{2}+Z_{p}^{2}}}{X_{p}}$	(20)

Here, *X_p_*, *Y_p_*, and *Z_p_* represent the end position of the continuum robot. Therefore, the two joint angles *β_1_* and *β_2_* within each fundamental bending unit of the bending joint can be derived as given in Equations (21) and (22):

$\beta_{1}=arctan(\cos\left( \theta_{segment} \right)*tan(\beta_{segment}))$	(21)

$\beta_{2}=arcsin(-sin \left( \theta_{segment} \right)*sin(\beta_{segment}))$	(22)

Based on the above equations, the length variations of the driving cables in the two segments of a fundamental bending unit are respectively given by:

$l_{11}=2\left( \frac{l_{link}}{\beta_{1}}-rsin\theta\right)*sin(\frac{\beta_{1}}{2})$	(23)

$l_{12}=2\left( \frac{l_{link}}{\beta_{1}}+rsin\theta\right)*sin(\frac{\beta_{1}}{2})$	(24)

$l_{13}=2\left( \frac{l_{link}}{\beta_{1}}+rcos\theta\right)*sin(\frac{\beta_{1}}{2})$	(25)

$l_{14}=2\left( \frac{l_{link}}{\beta_{1}}-rcos\theta\right)*sin(\frac{\beta_{1}}{2})$	(26)

$l_{21}=2\left( \frac{l_{link}}{\beta_{2}}+rcos\theta\right)*sin(\frac{\beta_{2}}{2})$	(27)

$l_{22}=2\left( \frac{l_{link}}{\beta_{2}}-rcos\theta\right)*sin(\frac{\beta_{2}}{2})$	(28)

$l_{23}=2\left( \frac{l_{link}}{\beta_{2}}+rsin\theta\right)*sin(\frac{\beta_{2}}{2})$	(29)

$l_{24}=2\left( \frac{l_{link}}{\beta_{2}}-rsin\theta\right)*sin(\frac{\beta_{2}}{2})$	(30)

Therefore, the total length of the *i*(*i=1, 2, 3, 4*) driving cable within this segment is expressed as:

$L_{i}=nl_{1i}+nl_{2i}+2nl_{d}$ (*i=1, 2, 3, 4*)	(31)

**Text S17: Compliance control strategy of a multi-joint continuum robot**

Figure S22 shows the compliance control strategy of a multi-joint continuum robot, designed to counteract the interference among bending joints. When a continuum robot integrated with E-armor comes into contact with the intestinal wall, the joint affected by the contact can be identified through tactile perception information. If contact occurs at only a single join, the system controls this joint to autonomously bend, avoiding continuous contact with the intestinal wall. It then corrects the posture of other joints to reduce the impact of joint coupling, to prevent the remaining joints from contacting the intestinal wall until the posture correction of the foremost joint is complete. If multiple joints are detected to be in contact simultaneously, the joint at the most distal end is selected as the current joint, and the aforementioned process is repeated. Ultimately, this allows the continuum robot to autonomously bend to avoid contact with the intestinal wall through tactile sensing information. This robot’s autonomous bending adjustment strategy starts with the most distal joint, adjusting its bending posture and progressively correcting the posture of joints towards the front, reducing the impact of joint coupling and avoiding excessive contact of each joint with the intestinal wall.

**Figure S22. Compliance control strategy of multi-joint continuum robot.**

**Text S18: Closed-loop shape control strategy and stability analysis for continuum robot**

1. **The closed-loop shape control strategy of the continuum robot**

From the kinematic analysis of the robot discussed earlier, it is understood that the bending shape of the continuum robot can be described by two parameters: the bending angle *β_segment_* and the twist angle *θ_segment_* of each bending joint. Controlling the bending shape of the continuum robot thus involves regulating these two parameters to achieve their desired values. As established in the preceding kinematic modeling and analysis, the bending angle *β_segment_* and twist angle *θ_segment_* of each bending joint exhibit a mapping relationship with the two joint angles *β_1_*, *β_2_* of the corresponding basic bending unit. These joint angles *β_1_*, *β_2_* are determined by changes in the driving wires, implying a direct mapping between the joint space and the actuation space. This mapping relationship can be expressed as:

$\boldsymbol{\beta}_{\boldsymbol{segment}}=f\left( \boldsymbol{q} \right)$	(32)

$\boldsymbol{\theta}_{\boldsymbol{segment}}=g\left( \boldsymbol{q} \right)$	(33)

$\boldsymbol{\beta}_{\boldsymbol{segment}}=\left[ \boldsymbol{\beta}_{\boldsymbol{segment}\mathbf{1}} \boldsymbol{\beta}_{\boldsymbol{segment}\mathbf{2}} \boldsymbol{\beta}_{\boldsymbol{segment}\mathbf{3}} \boldsymbol{\beta}_{\boldsymbol{segment}\mathbf{4}} \right]^{T}$	(34)

$\boldsymbol{\theta}_{\boldsymbol{segment}}=\left[ \boldsymbol{\theta}_{\boldsymbol{segment}\mathbf{1}} \boldsymbol{\theta}_{\boldsymbol{segment}\mathbf{2}} \boldsymbol{\theta}_{\boldsymbol{se}\boldsymbol{g}\boldsymbol{ment}\mathbf{3}} \boldsymbol{\theta}_{\boldsymbol{segment}\mathbf{4}} \right]^{T}$	(35)

In the equation above, ***q*** represents the actuation space variable, which corresponds to the length variation of the driving wires:

$\boldsymbol{q}=\left[ c_{1} c_{2}\cdots\cdots c_{16} \right]^{T}$	(36)

Based on the mapping relationship from joint space to actuation space, differentiating the change in driving wire lengths yields a functional relationship between the rate of change in driving wire lengths and the rate of change of joint variables. This relationship can be expressed as:

$\left[ \begin{matrix} \dot{\boldsymbol{\beta}_{\boldsymbol{segment}}} \\ \dot{\boldsymbol{\theta}_{\boldsymbol{segment}}} \end{matrix} \right]=\left[ \begin{matrix} \frac{\partial f}{\partial q} \\ \frac{\partial g}{\partial q} \end{matrix} \right]\left[ \dot{c_{1}} \dot{c_{2}}\cdots\cdots\dot{c_{16}} \right]^{T}$	(37)

The matrix of partial derivatives in the aforementioned functional relationship corresponds to the Jacobian matrix, which can be expressed as:

$\left[ \begin{matrix} \dot{\boldsymbol{\beta}_{\boldsymbol{segment}}} \\ \dot{\boldsymbol{\theta}_{\boldsymbol{segment}}} \end{matrix} \right]=J\left[ \dot{c_{1}} \dot{c_{2}}\cdots\cdots\dot{c_{16}} \right]^{T}$	(38)

From the above equation, we obtain:

$\left[ \dot{c_{1}} \dot{c_{2}}\cdots\cdots\dot{c_{16}} \right]^{T}=J^{+}\left[ \begin{matrix} \dot{\boldsymbol{\beta}_{\boldsymbol{segment}}} \\ \dot{\boldsymbol{\theta}_{\boldsymbol{segment}}} \end{matrix} \right]$	(39)

In the equation above, *J⁺* represents the pseudo-inverse of the Jacobian matrix *J*. Since *J* is not a square matrix and thus lacks an exact inverse, *J⁺* serves as the pseudo-inverse, which can be computed using the following formula:

$J^{+}={J^{T}\left( JJ^{T} \right)}^{-1}$	(40)

Therefore, based on the formulas above, the rate of change of the actuation space variables can be determined from the rate of change of the continuum robot’s joint space variables. Consequently, a corresponding controller is designed to regulate the robot’s bending shape. The developed bending shape control algorithm for the continuum robot is illustrated in the Figure S23.

**Figure S23. Flowchart of the bending shape control algorithm for the continuum robot.**

The controller for the shape control algorithm can be derived as:

$\dot{\boldsymbol{q}}={KJ}^{+}\mathbf{(}\boldsymbol{x}_{\boldsymbol{d}}\mathbf{-}\boldsymbol{x}\mathbf{)}$	(41)

Where, $x=[\beta_{segment} \theta_{segment}]^{T}$, $J^{+}=J^{T}\text{（}JJ^{T}\text{）}^{-1}$is the Moore-Penrose pseudoinverse of the Jacobian 𝐽, and

$K=\text{di}ag(k_{1},\ldots,k_{3}), k_{i}>0$ (42)

is chosen positive‐definite to ensure proper error feedback.

2. **Stability Analysis via Lyapunov Method**

Define the tracking error $\text{e}=\text{x}_{d}-\text{x}$, and consider the candidate Lyapunov function

$V(e)=\frac{1}{2}e^{T}e$ (43)

This function is non-negative and equals zero if and only if 𝑒 = 0, thus satisfying the conditions for a Lyapunov function.

From (38) and (41) we can obtain

$\mathop{e}\limits^{\cdot} =\mathop{x_{d}}\limits^{\cdot} -x=-x=-J\mathop{q}\limits^{\cdot} =-J(KJ^{+}e)=-JKJ^{+}e$ (44)

Hnece, the time derivative of (3.45) can be expressed as

$\mathop{V}\limits^{\cdot} =e^{T}\mathop{e}\limits^{\cdot} =-e^{T}JKJ^{+}e$ (45)

The gain matrix *K* is positive definite and carefully tuned such that the resulting matrix $JKJ^{+}$ is symmetric and positive definite. Hence, $-e^{T}JKJ^{+}\geq0$，leading to $\mathop{V}\limits^{\cdot} \leq0$. This control strategy ensures that the tracking error $\text{e}=\text{x}_{d}-\text{x}$ converges to zero, thereby enabling precise shape regulation of the continuum robot.

**Text S19: Compliance control strategy and autonomous navigation of a continuum robot integrated with E-armor in an intestine-shaped acrylic pipeline**

The contact situations of the continuum robot at six randomly selected time points during its crawling in the pipeline are captured (Figure S24a(i) to Figure S24a(vi)). First, Joint #1 enters the pipeline, continuously adjusting its posture to adapt to the pipeline’s shape, bending to the right (Figure S24a (i)). Once Joint #1 has fully entered, Joint #2 begins to follow suit, also bending to the right along the path previously taken by Joint #1 (Figure S24a (ii) to Figure S24a(iii)). As the continuum robot advances and reaches a corner in the pipeline, Joint #1 starts to bend to the left. At this point, Joint #2 has not yet arrived at the corner and continues bending to the right. Concurrently, Joint #3 begins its gradual entry into the pipeline, bending slightly to the right (Figure S24a(iv) to Figure S24a(vi)). In the entire process, bimodal signals of S1 to S8 and *R_1_* to *R_4_* from Joint #1 are shown (Figure S24b(i) and Figure S24b(ii)). Based on the tactile sensing code of S1 to S8 for each contact point Pi, the sequential order of contact events can be determined as P4-P4-P10-P4-P4-P4-P8-P8-P8-P8-P8+P12-P12-P4-P8-P8-P8-P2-P2-P6. The corresponding adaptive posture adjustments of 19 times can be observed (Figure S24b(ii)). From the stain information of *R_3_* and *R_4_*, it is clear that Joint #1 experiences a forward movement process of first bending to the right and then curving to the left. Similarly, the bimodal singles of S1 to S8 and *R_1_* to *R_4_* from Joints #2 and #3 are shown (Figure S24b(iii) to Figure S24b(vi)). It is observed that Joints #2 and #3 move through the pipeline after 40s and 52s delay of Joint #1. Then their postures sequentially adjusted based on the tactile and strain information. According to the encoding sequences, the contact points of Joints #2 and #3 are P6-P4-P8 and P6-P2, respectively. From the strain information, Joint #2 and #3 indicate significant and slight rightward bending movements, respectively, which is agree with their practical bending condition. A detailed depiction of the points of contact sensed by the E-armor units on each joint throughout the entire process, along with a tactile sensing code for all points, is provided (Figure S24c). As a result, the E-armor units effectively feedback tactile and strain sensing information during the robot crawling process, and the proposed compliance control strategy significantly improves the operational safety of the robot.

**Figure S24. Compliance control strategy and autonomous navigation of a continuum robot integrated with E-armor in an intestine-shaped acrylic pipeline.** a) Screenshots of the autonomous navigation of a continuum robot integrated with E-armor in an intestine-shaped acrylic pipeline. b) Bimodal signals of Joint #1, Joint #2 and Joint #3 during autonomous navigation in the pipeline. i) Tactile signals of Joint #1 during autonomous navigation in the pipeline. ii) Strain signals of Joint #1 during autonomous navigation in the pipeline. iii) Tactile signals of Joint #2 during autonomous navigation in the pipeline. iv) Strain signals of Joint #2 during autonomous navigation in the pipeline. v) Tactile signals of Joint #3 during autonomous navigation in the pipeline. vi) Strain signals of Joint #3 during autonomous navigation in the pipeline. c) Tactile sensing codes of Joint #1, Joint #2, and Joint #3.

**Figure S25. The preparation process of E-armor.** a) The preparation process of sensing belt. b) The preparation process of sensing ring.

**Table S9: Comparison of performance of triboelectric sensor arrays based on co-electrode strategy**

| Ref. | Materials | Stretchability | Stimuli | Points | Circuits | Simultaneous recognition of multiple points | Elimination of electrode crosstalk |
| --- | --- | --- | --- | --- | --- | --- | --- |
| ^[5]^ | Al strip and acrylic glass | Rigid | Sliding | 81 | 18 wires | Not available | Not available |
| ^[6]^ | PDMS and PDMS-PTFE | Flexible | Touch | 25 | 10 wires | Not available | Not available |
| ^[7]^ | PTFE@PMMA，FEP and nickel | Flexible | Sliding | 9 | 9 wires | Not available | Not available |
| ^[8]^ | Ecoflex, TPU, EVA copolymer sponge and PDMS | Flexible | Touch | 9 | 9 wires | Not available | Not available |
| **This work** | **Ecoflex, hydrogel, PTFE-Ecoflex and PA-Ecoflex** | **Flexible and stretchable** | **Touch** | **48** | **24 wires** | **Available** | Substrate separation and anti-static treatment |

**Table S10: Comparison of the performance of continuum robots integrated with E-skins**

| Ref. | Structure | Sensing mechanism | Modalities | Coverage distance/points | Circuits | Layout | Stretchability | Feedback |
| --- | --- | --- | --- | --- | --- | --- | --- | --- |
| ^[9]^ | Annular | Optoelectronic | Tactile | 170 mm/  51 points | 85 wires | Robotic exterior | Rigid | Not available |
| ^[10]^ | Array | Piezoelectric | Tactile | 60 mm/  5 points | 6  wires | Robotic interior | Rigid | Not available |
| ^[11]^ | Patch | Resistive | Tactile and strain | 240mm/  3 points | 14 wires | Robotic exterior | Flexible | Closed loop |
| ^[12]^ | Patch | Triboelectric | Tactile and proprioception | 0mm/  2 points | 6  wires | Robotic exterior | Flexible | Closed loop |
| ^[13]^ | Patch | Piezoresistive | Tactile | 30mm/  1 points | 2  wires | Robotic exterior | Flexible | Not available |
| ^[14]^ | Annular | Triboelectric | Tactile | 250 mm/  24 points | 24 wires | Robotic interior | Flexible | Closed loop |
| **This work** | **Array** | **Triboelectric and resistive** | **Tactile and strain** | **300 mm/**  **48 points** | **48 wires** | **Robotic interior** | **Flexible and stretchable** | **Closed loop** |

**Table S11: Autonomous posture adjustment strategy of continuous robots**

|  | **Ref.** | **Sensing mechanism** | **Modalities** | **Signal correlation** | **Adaptive posture adjustment** | **Robot oscillation suppression** |
| --- | --- | --- | --- | --- | --- | --- |
| [10] | | Ultrasonic | 1 | No | No | No |
|  | [11] | Strain, Pressure | 2 | Yes | Yes | No |
|  | [12] | Tactile, Proximity-tactile | 2 | No | Yes | No |
|  | [14] | Tactile | 1 | No | No | No |
|  | [15] | Tactile, Strain | 2 | Yes | Yes | No |
|  | [16]  **This work** | Tactile, Strain  Tactile, Strain | 2  2 | Yes  Yes | No  Yes | No  Yes |

**Reference:**

[1]	M. Esaki, T. Yamamura, M. Nakamura, K. Maeda, T. Sawada, Y. Mizutani, E. Ishikawa, H. Suzuki, T. Kuno, K. Yamada, I. Hasegawa, T. Ishikawa, N. Kakushima, K. Furukawa, E. Ohno, H. Kawashima, Y. Hirooka, M. Fujishiro, *Digestion* **2021**, *102*, 701.

[2]	G. Ciasca, M. Papi, E. Minelli, V. Palmieri, M. De Spirito, *World J Gastroenterol* **2016**, *22*, 7203.

[3]	L.-D. Chen, W. Wang, J.-B. Xu, J.-H. Chen, X.-H. Zhang, H. Wu, J.-N. Ye, J.-Y. Liu, Z.-Q. Nie, M.-D. Lu, X.-Y. Xie, *Radiology* **2017**, *285*, 279.

[4]	D. Shalon, R. N. Culver, J. A. Grembi, J. Folz, P. V. Treit, H. Shi, F. A. Rosenberger, L. Dethlefsen, X. Meng, E. Yaffe, A. Aranda-Díaz, P. E. Geyer, J. B. Mueller-Reif, S. Spencer, A. D. Patterson, G. Triadafilopoulos, S. P. Holmes, M. Mann, O. Fiehn, D. A. Relman, K. C. Huang, *Nature* **2023**, *617*, 581.

[5]	C. Bao Han, C. Zhang, X. H. Li, L. Zhang, T. Zhou, W. Hu, Z. Lin Wang, *Nano Energy* **2014**, *9*, 325.

[6]	Y. Lin, S. Duan, D. Zhu, Y. Li, B. Wang, J. Wu, *Adv. Intell. Syst.* **2023**, *5*, 2100120.

[7]	Y. Ren, G. Liu, H. Yang, T. Tong, S. Xu, L. Zhang, J. Luo, C. Zhang, G. Xie, *Nano Energy* **2020**, *68*, DOI 10.1016/j.nanoen.2019.104303.

[8]	S. Lv, X. Zhang, T. Huang, H. Yu, M. Zhu, *Nano Energy* **2021**, *89*, 106476.

[9]	Y. Noh, S. Han, P. Gawenda, W. Li, S. Sareh, K. Rhode, *IEEE Sens J* **2020**, *20*, 3487.

[10]	Z. Yin, Y. Hong, X. Sun, Z. Shen, Y. Zhang, F. Ju, B. W. Drinkwater, in *2022 IEEE/RSJ Int. Conf. Intell. Robots Syst. (IROS)*, IEEE, **2022**, pp. 5945–5950.

[11]	H. Yan, Y. Wang, W. Shen, F. Li, G. Gao, T. Zheng, Z. Xu, S. Qian, C. Chen, C. Zhang, G. Yang, T. Chen, *Adv. Funct. Mater.* **2022**, *32*, 2203241.

[12]	W. Liu, Y. Duo, J. Liu, F. Yuan, L. Li, L. Li, G. Wang, B. Chen, S. Wang, H. Yang, Y. Liu, Y. Mo, Y. Wang, B. Fang, F. Sun, X. Ding, C. Zhang, L. Wen, *Nat. Commun.* **2022**, *13*, 5030.

[13]	T.-H. Chang, Y. Tian, C. Li, X. Gu, K. Li, H. Yang, P. Sanghani, C. M. Lim, H. Ren, P.-Y. Chen, *ACS Appl. Mater. Interfaces.* **2019**, *11*, 10226.

[14]	Y. Sun, H. Li, C. Hou, W. Shen, H. Liu, T. Chen, H. Ren, X. Zuo, Y. Li, in *2021 21st Int. Conf.* *Solid-State Sens., Actuators Microsys. (Transducers)*, IEEE, **2021**, pp. 329–332.

[15]	P. Wang, Z. Xie, W. Xin, Z. Tang, X. Yang, M. Mohanakrishnan, S. Guo, C. Laschi, *Nat. Commun.* **2024**, *15*, 9978.

[16]	G. Jin, Y. Sun, J. Geng, X. Yuan, T. Chen, H. Liu, F. Wang, L. Sun, *Nano Energy* **2021**, *84*, 105896.
